# Long, Noncoding RNA Dysregulation in Glioblastoma

**DOI:** 10.3390/cancers13071604

**Published:** 2021-03-31

**Authors:** Patrick A. DeSouza, Xuan Qu, Hao Chen, Bhuvic Patel, Christopher A. Maher, Albert H. Kim

**Affiliations:** 1 Department of Neurological Surgery, Washington University School of Medicine in St. Louis, St. Louis, MO 63110, USA; pdesouza@wustl.edu (P.A.D.); quxuan@wustl.edu (X.Q.); chenhao@wustl.edu (H.C.); bhuvic.patel@wustl.edu (B.P.); 2 Department of Internal Medicine, Washington University School of Medicine in St. Louis, St. Louis, MO 63110, USA; christophermaher@wustl.edu; 3 Department of Neuroscience, Washington University School of Medicine in St. Louis, St. Louis, MO 63110, USA; 4 Department of Biomedical Engineering, Washington University School of Medicine in St. Louis, St. Louis, MO 63110, USA; 5 McDonnell Genome Institute, Washington University School of Medicine in St. Louis, St. Louis, MO 63110, USA; 6 Siteman Cancer Center, Washington University School of Medicine in St. Louis, St. Louis, MO 63110, USA

**Keywords:** noncoding RNA, miRNA, lncRNA, glioblastoma, heterogeneity, RNAi

## Abstract

**Simple Summary:**

Developing effective therapies for glioblastoma (GBM), the most common primary brain cancer, remains challenging due to the heterogeneity within tumors and therapeutic resistance that drives recurrence. Noncoding RNAs are transcribed from a large proportion of the genome and remain largely unexplored in their contribution to the evolution of GBM tumors. Here, we will review the general mechanisms of long, noncoding RNAs and the current knowledge of how these impact heterogeneity and therapeutic resistance in GBM. A better understanding of the molecular drivers required for these aggressive tumors is necessary to improve the management and outcomes of this challenging disease.

**Abstract:**

Transcription occurs across more than 70% of the human genome and more than half of currently annotated genes produce functional noncoding RNAs. Of these transcripts, the majority—long, noncoding RNAs (lncRNAs)—are greater than 200 nucleotides in length and are necessary for various roles in the cell. It is increasingly appreciated that these lncRNAs are relevant in both health and disease states, with the brain expressing the largest number of lncRNAs compared to other organs. Glioblastoma (GBM) is an aggressive, fatal brain tumor that demonstrates remarkable intratumoral heterogeneity, which has made the development of effective therapies challenging. The cooperation between genetic and epigenetic alterations drives rapid adaptation that allows therapeutic evasion and recurrence. Given the large repertoire of lncRNAs in normal brain tissue and the well-described roles of lncRNAs in molecular and cellular processes, these transcripts are important to consider in the context of GBM heterogeneity and treatment resistance. Herein, we review the general mechanisms and biological roles of lncRNAs, with a focus on GBM, as well as RNA-based therapeutics currently in development.

## 1. Noncoding RNAs Are Transcribed from a Large Proportion of the Genome

### 1.1. Noncoding RNA Nomenclature and Biogenesis

Of the 3 billion nucleotides that make up the human genome, only 2% produce transcripts that code for proteins. However, transcription occurs across more than 70% of the human genome, producing many noncoding transcripts of various sizes [1,2,3,4]. Although a large fraction of these transcripts may not be functional, more than half of currently annotated genes produce noncoding RNAs (ncRNAs) [5]. Noncoding RNAs are divided into two main categories based on their size. A 200 bp size threshold was adopted due to the biochemical fractionation properties of RNA, thus separating long ncRNAs (lncRNAs) from small ncRNAs (sRNAs), which include tRNAs, rRNAs, and small, nuclear and nucleolar RNAs [6,7].

**Figure 1:**
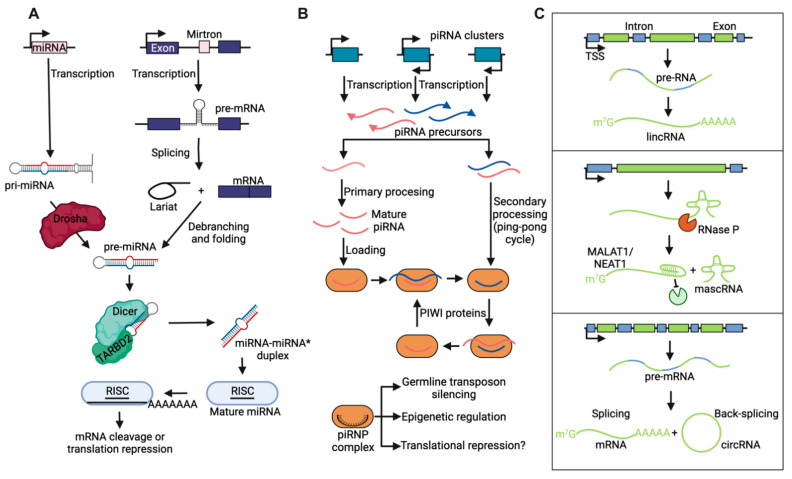
Biogenesis of small and long ncRNAs. (**A**) miRNAs are transcribed at independent loci (primary miRNA [pri-miRNA]) or together with host protein-coding genes (mirtrons). After processing by the Drosha complex or lariat-debranching enzymes, respectively, precursor miRNAs (pre-miRNAs) are shuttled to the cytoplasm for further processing by Dicer and TAR RNA-binding protein 2 (TARBP2). When two mature miRNAs originate from opposite arms of the same pre-miRNA, one mature species is typically more abundant than that derived from the opposite arm, in which case, an asterisk indicates the low abundant species. Following generation of mature miRNAs, which are loaded onto the RNA-induced silencing complex (RISC), miRNAs function through degradation of protein-coding transcripts or translational repression. (**B**) PIWI-interacting RNAs (piRNAs) are mostly expressed as ssRNAs from mono- or bidirectional clusters. Additional piRNAs may be produced through a PIWI-protein-catalyzed amplification loop (“ping-pong cycle”) via sense and antisense intermediates. The PIWI ribonucleoprotein (piRNP) complex functions in transposon repression through target degradation and epigenetic silencing. Roles of the piRNP complex in translation repression, if any, remain unknown. (**C**) (Top) Long intergenic ncRNAs (lincRNAs) are transcribed by Poll II from intergenic regions (>20 kb from closest protein-coding gene), and spliced, capped, and polyadenylated. (Middle) MALAT1 and NEAT1 are well-studied, highly conserved lncRNAs that are processed by RNase P and stabilized by U-A-U triple helix structures at their 3′ ends. Their 3′-end products are further processed to form MALAT1-associated small cytoplasmic RNA (mascRNAs), which are ~60 nt in length and have unknown functions. (Bottom) Circular RNA (circRNAs) are produced from back-slicing circularization of exonic pre-mRNAs. During splicing, pre-mRNAs are spliced into linear, mature mRNAs or back-spliced into circRNAs.

sRNAs are cleavage products of endogenous or exogenous primary transcripts and often target the recruitment of other proteins in trans, at a physically distinct location from the locus of synthesis (Figure 1A,B) [8]. The most frequently studied groups of sRNAs are 20–30 bp in length and associate with Argonaute (Ago) family proteins [9], although 30–60 bp precisely processed Y RNA and tRNA fragments have also been detected—for instance, in microvesicles and exosomes isolated from patient-derived glioblastoma cell cultures [10]. Based on their mechanisms of biogenesis, these well-studied sRNAs have been divided into classes, among which we will briefly describe microRNAs (miRNAs) and PIWI-associated RNAs (piRNAs). miRNAs are cleavage products of endogenous hairpin ncRNAs by Drosha and Dicer proteins. miRNAs are loaded onto a ribonucleoprotein complex that includes Ago proteins and guide the RNA-induced silencing complex (RISC) to complementary transcripts (Figure 1A) [11]. RISC mediates posttranscriptional gene silencing by triggering transcript degradation or inhibiting the translation of the complementary mRNAs. piRNAs are associated with the PIWI clade of Ago proteins (Figure 1B). PIWI-piRNA complexes are crucial for protecting genomes against instability by repressing transposon activity via transcriptional and/or posttranscriptional silencing, especially in the germline, where cells undergo rapid changes in genome accessibility and transcription dynamics [12,13].

The majority of ncRNA species are lncRNAs, which bear some resemblance in biogenesis and processing to mRNAs, except that traditionally no protein has been detected or predicted as a result of their production (Figure 1C) [14,15,16]. In recent years, however, functionally annotated lncRNAs, such as LINC-PINT, have demonstrated short open reading frames that express small peptides with regulatory function, indicating dual RNA-peptide activity from “noncoding” genomic loci [17,18]. This has informed nomenclature as efforts to catalogue the noncoding genome have rapidly accelerated. Still, lncRNA species can be subdivided based on their proximity to protein coding genes and unique features of biogenesis that influence their final structure (Figure 1C) [19,20,21]. For example, intergenic lncRNAs are transcribed from loci >20 kbp away from any protein-coding gene and include independently transcribed loci as well as RNAs transcribed from enhancers that aid the activation of looped promoters. Well-described lncRNAs MALAT1 and NEAT1 are processed by RNase P and stabilized by U-A-U triple helix structures at their 3′ ends. Finally, circular RNAs are produced by back-splicing circularization of “exons” from pre-mRNAs and remain stably retained in the nucleus without polyadenylation signaling.

### 1.2. Evolutionary Conservation of Noncoding Transcripts

Generally, lncRNAs are less evolutionarily conserved at the sequence-level than mRNAs, contain fewer “exons” after splicing, and are more likely localized in the nucleus [22,23,24]. Nuclear lncRNAs have been historically investigated in the contexts of their gene neighborhoods for acting as platforms that assemble regulatory complexes in *cis*, although mechanisms in *trans* at physically distant genomic loci are becoming increasingly appreciated, for example by nontraditional base pairing and/or docking of chromatin-associated ribonucleoprotein complexes [25,26,27]. Specific and more widespread pleotropic functions have been increasingly ascribed to each class of RNA molecules, including various architectural and/or gene regulatory roles in different cellular compartments [23]. While many RNAs are unlikely to act alone and instead interact with specific RNA binding proteins, previously described DNA binding proteins, such as the GBM master transcription factor SOX2, have demonstrated RNA binding capabilities, increasing the potential repertoire of molecular interactions in tumor cells for diverse specialized functions [28,29].

The lack of evolutionary conservation in many lncRNAs sequences has spurred speculation that many transcripts of low abundance are simply noise, perhaps reflecting a degree of promiscuous action of the transcription machinery sampling open chromatin regions [23,30]. However, it is clear that many lncRNAs have specialized and cell context-specific functions beyond contributing to general transcriptional tone. Regardless of the scope of a given lncRNA’s activity, it has become increasingly apparent that the conservation of secondary structure is a stronger driving force for noncoding transcriptome evolution than the conservation of primary sequence. Primary sequence relationships between lncRNAs were deconstructed to evaluate similarity based on the abundance of short motifs called k-mers [31]. Transcripts with related function often had similar k-mer profiles despite a lack of linear homology, and k-mer profiles correlated with protein binding partners and with subcellular localization. This supports the importance of binding motifs, patterns, and partners, for dictating the local thermodynamic environments that define epigenetic activity and a need for better understanding of the molecular “language” used in particular by malignant cells, which we hypothesize rely on epigenetic flexibility [32]. Furthermore, even ‘junk’ transcripts reflecting biological noise may provide raw material for the evolution of functional noncoding transcripts by nonadaptive mechanisms, such as constructive neutral evolution [33]. For example, although chromatin remodeling by RNA polymerase II likely evolved under the selective pressure to suppress spurious transcription that originates within gene bodies, this process can be co-opted to downregulate endogenous genes [34]. Under weak selective pressures, transcription binding sites and cryptic transcriptional start sites in intergenic regions persistently emerge and vanish, so long as they do not perturb the equilibrium that drives an organism’s fitness. The high prevalence of these sites in the genome, sustained by their frequent appearance and disappearance over time, increases the chances that beneficial transcriptional regulatory events arise.

### 1.3. Long, Noncoding RNAs in the Central Nervous System

Of the tens of thousands of lncRNA genes annotated from the GENCODE and ENCODE projects, 40% (anywhere from 4000–20,000 lncRNA genes) are expressed specifically in the brain [24]. This is at least two times more than any other organ, including the testes, although the latter organ demonstrates the highest expression levels of lncRNAs despite having a smaller repertoire. The number of brain-specific lncRNAs is strikingly large given the human genome contains approximately 20,000–25,000 protein-coding genes in total and around 2500 miRNAs. Although the protein and miRNA expression profiles of the central nervous system are more diverse than other organs, only a subset of these are specific to the nervous system [35,36]. The expression of lncRNAs is dynamically regulated during neural development and in response to neuronal activity [37]. Specific lncRNA expression is often highly restricted to particular brain regions and it has been suggested that lncRNAs provide more information about cell type identity during mammalian cortical development than protein-coding genes [38,39,40]. This implies an intimate connection and parallel diversity between lncRNAs and fate commitment in the neuroectodermal lineage as a means of coordinating spatially distinct, yet synchronous responses with contacts and processes. These dynamics and region-specific expression patterns are coordinated by cell-intrinsic or signal-dependent transcription factors as well as well-defined chromatin dynamics at lncRNA loci [41,42]. This raises the possibility that an intricate, highly regulated noncoding RNA axis evolved for highly specialized cellular functions, such as in the brain and testes. The testes express trans-acting regulatory lncRNAs required for the complex, intricate process of spermatogenesis [43]. Rigorous investigation about whether the specific repertoires of noncoding transcriptomes have any relation to the common immune-privileged status of these organs has only recently emerged [44,45]. Access to a large portion of the noncoding genome and transcriptome in highly specialized, immune-privileged tissues thus represent unexplored mechanisms that may contribute to the accelerated Darwinian evolution in malignantly transformed cells originating in these organs.

## 2. General Mechanisms of lncRNAs

**Figure 2:**
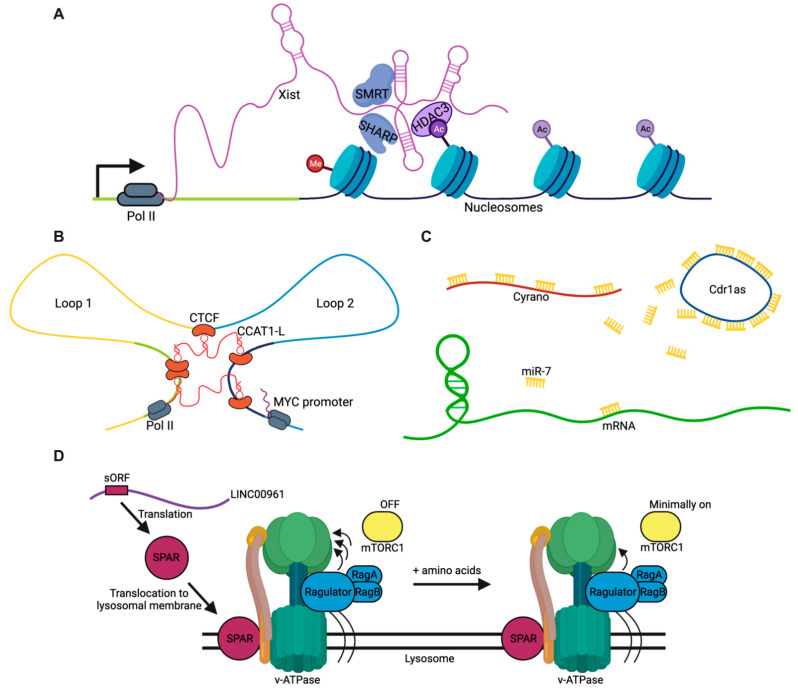
Mechanisms of lncRNAs. (**A**) lncRNAs can act as guides, such as Xist which recruits HDAC1-associated protein (SHARP), silencing the mediator for retinoid and thyroid hormone receptor (SMART), and HDAC3 to inactivate an X chromosome [48,49]. (**B**) lncRNAs can act as scaffolds, such as CCAT1-L, which accumulates in *cis* to regulate chromatin looping between enhancers and the *MYC* promoter [50]. (**C**) lncRNAs can act as sponges, such as in the ceRNA regulatory network where the lncRNA Cyrano triggers degradation of miR-7 and prevents it from repressing its target RNAs including the circRNA Cdr1as [51]. (**D**) Some previously annotated lncRNAs may encode peptides, such as LIN00961 whose small open reading frame (sORF) is translated into the small regulatory polypeptide of amino acid response (SPAR) that binds to lysosomal ATPase and prevents the dissociation of Ragulator upon amino acid stimulation resulting in minimal mTORC1 activation [52].

Since their discovery in recent decades, many studies have begun dissecting how lncRNAs are required for important molecular and cellular functions [46]. The following roadmap outlines the major known functions of currently annotated lncRNAs, with the recognition that additional as-of-yet unknown mechanisms likely exist for these transcripts. This roadmap can be broadly divided into four major categories: guides, scaffolds, sponges, and peptides (Figure 2). The broadest and best-documented category describes how lncRNAs act as “guides” to direct heteromeric macromolecule interactions, such as between proteins and DNA or lipids and proteins (Figure 2A). Similarly, lncRNAs can also act as scaffolds to facilitate interactions between macromolecules of the same class, such as between p53 and MDM2 proteins [47], or for defining looped neighborhoods of distant genomic loci (Figure 2B). More recently, there has been increased interest in the sponging abilities of lncRNAs to sequester miRNAs and proteins, thus preventing their actions without necessarily degrading them to form competitive, endogenous networks (ceRNA networks) that regulate temporal dynamics of molecular stability and inhibition (Figure 2C). Finally, as mentioned earlier, some lncRNA species possess small open reading frames that encode short peptides with regulatory function (Figure 2D), motivating a critical reassessment of previously annotated “noncoding” regions of the genome and updated nomenclature for future classification.

### 2.1. Guides

The mechanism of lncRNAs best represented in current literature describes their function to facilitate interactions between macromolecules of differing classes. This is exemplified by long intergenic noncoding RNA for kinase activation (LINK-A), which interacts with the AKT pleckstrin homology domain and PIP_3_ at the single-nucleotide level, enabling their interaction and enzymatic activation. AKT hyperactivation in a LINK-A-dependent manner leads to tumorigenesis and resistance to targeted inhibitors [53]. In the nucleus, the guide-mediated formation of ribonucleoprotein complexes is a particularly prevalent and well-described mechanism of lncRNAs to trigger epigenetic changes (Figure 2A). In mouse trophoblast stem cells, the AIRN and KCNQ1OT1 lncRNAs induce polycomb repressive complex (PRC)-dependent chromatin modifications over multimegabase domains [54]. Thus, CpG islands that independently recruit PRCs can interact with lncRNAs and their associated proteins through three-dimensional space to nucleate the spread of PRCs in lncRNA-targeted regions. This targeting occurs through common mechanisms dependent upon chromatin environment surrounding these noncoding genes and their transcript abundances. Furthermore, it has been demonstrated that PRC2 requires RNA binding for chromatin localization in human pluripotent stem cells and defining cellular state [55].

The crosstalk between lncRNAs and well-described chromatin-associated regulatory complexes is further supported by recently published data. For example, the lncRNA SWINGN influences the ability of the SWI/SNF complexes to drive the epigenetic activation of specific promoters via SMARCB1-dependent activity in topologically organized regions [56]. Still, the exact biophysical characteristics that dictate these means of regulation have yet to be fully characterized. As previously mentioned, the structural domains of lncRNAs demonstrate stronger conservation than the underlying primary sequences. The systematic deletion of NEAT1 portions revealed modular domains important for RNA stability, isoform switching, and paraspeckle assembly though phase separation [57]. We are continually gaining appreciation for newly discovered stereotyped conformations found in noncoding species such as the pseudoknot, a secondary structure containing two stem-loop structures in which half of one stem is intercalated between the two halves of another stem. Pseudoknots represent some of the most conserved elements in all of evolution, especially that found in RNase P, and the conserved pseudoknot region of the lncRNA MEG3 is essential for stimulation of the p53 pathway [58].

### 2.2. Scaffolds

In addition to facilitating heteromeric macromolecular interactions, the length and structural components of lncRNAs confer them with scaffolding functions between macromolecules of the same class. Several binding studies in immortalized lung and colon cancer cell lines indicated the formation of a ternary complex between the lncRNA SENEBLOC (SBLC) and the proteins p53 and MDM2 [47]. Notably, the interaction between p53 and MDM2 by coimmunoprecipitation was substantially reduced when SBLC was silenced or when samples were pretreated with RNase. Binding assays demonstrated that SBLC binds p53 through its C-terminal regulatory domain, whereas SBLC binds to the central acidic region of MDM2. This suggests that SBLC drives the association between p53 and MDM2, which serves to facilitate p53 degradation.

Scaffolding also plays a role in defining chromatin architecture by facilitating the looping of distant genomic loci into close proximity for transcription regulation (Figure 2B). CCAT1-L is transcribed specifically in human colorectal cancers from a locus 515 kb upstream of *MYC*. This lncRNA promotes long-range chromatin looping for regulating transcription at the *MYC* locus [50]. It is transcribed from a locus located within a strong superenhancer region that is spatially close to the *MYC* gene. Knockdown of CCAT1-L reduced long-range interactions between the *MYC* promoter and its enhancers. This is mediated by CTCF interactions, which modulate chromatin conformation at these loop regions.

### 2.3. Sponges

While lncRNAs may interact with a variety of molecules to form complexes with important regulatory function, they may also bind molecules for targeted inhibition and help titrate cellular dosage. For example, a network of four ncRNAs act in the mammalian brain as a competitive, endogenous network to dampen neuronal activity [51]. The lncRNA Cyrano utilizes a landing pad of extensive miR-7 binding sites to trigger destruction of this miRNA (Figure 2B). By reducing miR-7 levels, Cyrano enables the accumulation of the miR-7-targeted Cdr1as, a circular RNA known to regulate neuronal activity. Without Cyrano, accumulation of miR-7 triggers cytoplasmic destruction of Cdr1as in neurons, in part through enhanced silencing by a second miRNA, miR-671.

However, these competitive, endogenous networks are not limited to RNA–RNA interactions. The noncoding RNA activated by DNA damage (NORAD) is a highly conserved and abundant lncRNA whose guide-mediated function in the topoisomerase complex is crucial for genome stability [59]. Remarkably, a sponging mechanism also contributes to dramatic aneuploidy in previously karyotypically stable cell lines upon the inactivation of NORAD [60]. NORAD maintains genomic stability by sequestering Pumilio-Fem3-binding factor (PUF) proteins, which bind with high specificity to sequences in the 3′ UTRs of target mRNAs through their PUMILIO homology domains. In the absence of NORAD, PUMILIO proteins, PUM1 and PUM2, drive chromosomal instability by hyperactively repressing mitotic, DNA repair, and DNA replication factors.

### 2.4. Peptides

Matsumoto et al. first functionally characterized a novel peptide encoded by the lncRNA LINC00961 that localizes to the late endosome/lysosome and interacts with the lysosomal v-ATPase to negatively regulate mTORC1 activation (Figure 2D) [52]. This regulation of mTORC1 is specific to activation by amino acid stimulation, and downregulation occurs in skeletal muscle upon acute injury to promote muscle regeneration in a tissue-specific manner. In GBM, an 87-amino-acid peptide encoded by the circular form of long intergenic non-protein-coding RNA p53-induced transcript (LINC-PINT) suppresses cell proliferation in vitro and in vivo [18]. This peptide directly binds the polymerase associated factor complex and prevents the transcriptional elongation of multiple oncogenes. The expression of LINC-PINT RNA and peptide are decreased in GBM compared with expression in normal tissue. The investigation of peptide-encoding lncRNAs is rapidly evolving with genomewide efforts to better characterize regulatory functions of “noncoding” genomic loci.

### 2.5. Considering Structural Determinants of lncRNA Function

Although this classification scheme encompasses the large majority of currently described lncRNA mechanisms, there are lncRNA functions that do not fit well into this classification scheme. For instance, the lncRNA for calcium-dependent kinase activation (CamK-A) binds the Ca^2+^/calmodulin-dependent kinase PNCK, which releases the auto-inhibition of PNCK and enhances its autophosphorylation and kinase activity in a dose-dependent manner [61]. Thus, the presence of lncRNAs in various cellular compartments and complexes is important for defining the structural environment in normal cells and in pathologic processes [62,63].

More recently, RNA G-quadruplex (RG4) structures have emerged as intriguing conformations because of their unique structural properties and roles in epigenetic mechanisms and cellular functions. Interestingly, their dysregulation has been proposed to have an impact on human disease, including cancer [64]. RG4s are extremely stable structures formed by stacking of two or more G quartets, each composed of four guanines interacting via Hoogsteen bonding. In GBM, the RNA binding proteins hnRNP H and F synergize with the RNA helicase DHX36 to bind unfolded RG4s in the cytoplasm to mediate translation-linked genomic instability and therapy resistance [65]. Although we are only starting to dissect the functions of these conformations in noncoding transcripts, one of the best-described lncRNAs, H19, contains a 5′ RG4 that facilitates binding of Sp1 or E2F1 to regulate its own expression [66]. More work is needed to understand how prevalent these modular structures are among lncRNAs, their roles in lncRNA function, and their contribution to disease processes.

## 3. Glioblastoma Is an Aggressive, Fatal Brain Tumor

Gliomas are the most prevalent type of malignant primary brain tumor, accounting for 78 percent of malignant brain tumors [67]. They are thought to arise from progenitor cells in the adult CNS or alternatively through the dedifferentiation of more mature cell types [68,69]. Grade IV gliomas, or glioblastomas (GBMs), are the most aggressive type of glioma, as highlighted by their ability to evade standard therapies and recur rapidly, resulting in an average survival of only 17 months [67]. These tumors tend to occur after at a median age 65 years and occur at a higher incidence in men than women.

The evolutionary trajectories of GBM can be divided into at least two major phases: a longer initial tumorigenesis phase and a recurrence phase separated by therapeutic intervention [70,71]. Tumorigenesis consists of the initiation events and subclinical growth leading up to clinical detection, while recurrence is characterized by therapeutic resistance and more rapid tumor growth. With few exceptions, recurrent tumors generally do not demonstrate substantial genetic selection and often resemble their primary tumor [72,73]. The structural variants, chromosome 7 gain, 9 loss, and 10 loss, are considered the earliest initiating events in GBM, and are followed by mutations in the associated genes, EGFR, CDKN2A, and PTEN, respectively [70,71]. These alterations further select for mutations in familiar cancer-related genes, such as TERT (promoter region), NF1, TP53, PDGFRA, CDK4, and EGFR, which occur at a high frequency among GBM patients and are considered required events that drive tumorigenesis. Notably, mutations in IDH1 and IDH2 are common driver mutations in low grade gliomas, a subset of which can progress to grade IV astrocytomas, are distinct from *IDH* wildtype GBMs, and represent a parallel disease process with likely alternative mechanisms of evolution [72].

Temozolomide (TMZ), an alkylating agent, is the current primary chemotherapeutic agent used to treat GBM, and is combined with radiotherapy for standard of care [74]. Although the investigation of new treatment paradigms in GBM has informed our understanding of the epigenetic fitness GBM cells possess for therapeutic resistance, we will briefly describe the molecular response to the current standard of care chemotherapeutic, TMZ. TMZ prevents the G2/M transition during proliferation and ultimately leads to the initiation of apoptosis. It causes cytotoxicity by methylating guanine at the O-6 site, which causes the addition of thymine instead of cytosine, leading to cell death. Concomitant therapy with both TMZ and radiation improved overall median survival by 2.5 months in newly diagnosed adult GBM patients as compared with those treated with radiation alone [74]. Alterations in DNA repair mechanisms, especially in the O-6-methylguanine-DNA methyltransferase (MGMT) enzyme, are thought to contribute to initial TMZ resistance and tumor relapse. MGMT reverses the TMZ effect by removing the methyl attached to the O-6 guanine residue, thereby leading to the failure of therapy [75,76]. However, even patients with a methylated MGMT promoter—and thus little to no expression of MGMT—eventually recur following standard of care treatment. In part, the development of effective therapies has been limited by an incomplete understanding of how genetic and epigenetic aberrations coordinate adaptive gene expression programs and functions to achieve treatment resistance.

## 4. Tumor Heterogeneity and Therapeutic Resistance in GBM

### 4.1. Defining Transcriptional Subtypes in GBM

Recent rapid advances in sequencing technologies have facilitated a better understanding of the complex disease processes underlying GBM. Early bulk gene expression profiling in GBM established three major subtypes that broadly categorized patient tumors based on dominant transcriptional programs termed classical, proneural, and mesenchymal [77,78] However, the classification of tumors by these subtypes only minimally stratifies prognosis on a proneural to mesenchymal axis with no robust differences in therapeutic response to date. The advent of single-cell transcriptome sequencing has revealed how transcriptional subtypes vary even within individual GBM tumors, with single cells in one tumor exhibiting different transcriptional subtypes [79]. Thus, a more holistic categorization of tumor cells beyond gene expression should capture how genetic background generates the spectrum of epigenetic fitness capabilities each cell possesses for survival, adaptation, and disease progression.

Single-cell transcriptomic studies in recent years have provided an unprecedented level of resolution dissecting the gene expression heterogeneity that characterizes GBMs. Malignant cell populations are broadly composed of four transcriptional “states” resembling those of native astrocytes, oligodendrocytes, less committed progenitor cells that normally give rise to both of these former cell types, and a mesenchymal-like state that has specific immune cell interactions in the tumor microenvironment [80]. Unlike normal lineage commitment, an adaptive plasticity exists between these developmentally related states in GBM that allows cells to transition between gene expression programs.

### 4.2. Neurodevelopmental Signatures in GBM Heterogeneity and Therapeutic Resistance

Utilizing a neurodevelopmental framework, Dirks and colleagues first introduced the concept of an epigenetically determined developmental hierarchy with a subset of neural progenitorlike cells within the tumor cell population, GBM stem cells (GSCs), sitting at the apex of that hierarchy [81,82]. GSCs are thought to drive tumor growth and contribute to therapy resistance [68]. Single cell RNA analysis of proliferating GBM GSCs cells were found to reside on a single axis of variation, ranging from proneural to mesenchymal, with mesenchymal cells as the progenitors of proneural cells [83]. A more recent single cell transcriptomics study found that single cell heterogeneity in GBM is in part due to a conserved neural trilineage cancer hierarchy centered around glial progenitorlike cells [84]. RNA velocity and pseudotime analyses revealed commitment of these glial progenitorlike cells toward oligdendrocyte expression patterns, neuronal cancer cell types, and a hybrid astrocytic-mesenchymal lineage. Thus, the stem cell hierarchy within GBM cell populations, in part, resembles the program dynamics during normal brain development. Within this heterogeneous GSC compartment is a particularly invasive subpopulation that transcriptionally resembles outer radial glia, a fetal cell type that expands the stem cell niche in normal human cortex. This GSC subpopulation undergoes characteristic mitotic somal translocation behavior previously observed in normal neural development, suggesting a reactivation of developmental programs in GBM [85].

The upregulation of primitive neurodevelopmental programs has also been shown to confer GSC populations with the ability to reversibly transition to a slow-cycling treatment-resistant state in response to targeted kinase inhibitors [86]. After drug treatment, the upregulation of Notch signaling is accompanied by widespread redistribution of repressive histone methylation. As such, these resistant GSCs upregulate, and are dependent on, the histone demethylases KDM6A/B. Slow-cycling cells with high Notch activity and histone demethylase expression are present in primary GBM before treatment, suggesting the potential for adaptive chromatin remodeling during recurrence. How exactly these resistant cells may be activated and bestowed with plasticity that promotes the sampling and co-opting of adaptive molecular mechanisms for recurrence remains largely unknown. It also remains unknown how a spectrum of plasticity could be dynamically regulated in GSCs and tumor cells resembling a more lineage-committed differentiation state.

### 4.3. Influence of Tumor Microenvironment

One striking example of how the tumor environment may influence the epigenetic fitness of GBM tumor cells is the discovery that cancer cells form interactions and even synapses with nonmalignant, cancer-associated neurons to contribute to malignant growth, a burgeoning referred to as cancer neuroscience [87]. In the adult brain, neuronal activity induces neuroglial stem and progenitor cell proliferation and migration via excitatory synapses [88]. Combined ultrastructural and electrophysiological studies identified glutamatergic synapses between neuronal axon terminals and postsynaptic glioblastoma cells [89]. In xenograft models, signaling via neuron-glioma synapses promoted tumor growth and invasiveness, whereas inhibiting synaptic transmission had cytostatic effects. Furthermore, the synaptic protein neuroligin-3 (NLGN3) was identified as a mitogen secreted from active neurons that was necessary and sufficient to promote robust GBM cell proliferation [90]. A deeper understanding of the molecular mechanisms that confer GBM cells with epigenetic fitness is required to overcome current barriers in therapeutic innovation.

In addition to the complex synaptic landscape at the tumor–brain interface, the access of most drugs to the tumor site is limited by physiological barriers, including the blood–brain barrier (BBB) and blood–tumor barrier (BTB) [91]. The relatively less understood and unique immune ecosystem of the brain also poses challenges for the immune-mediated destruction of cancer cells, especially given the suppressive nature of the tumor microenvironment [92]. The current barriers to therapeutic innovation in GBM thus motivate a closer examination of the local environmental cues that confer GBM cells with epigenetic fitness as well as the mechanisms that inhibit drug and immune cell access in GBM. The role of the noncoding transcriptome in these processes remains to be explored.

## 5. Noncoding Aberrations Are Understudied Molecular Players in GBM

### 5.1. Somatic Drivers and Structural Variants in lncRNAs

Identifying genetic drivers in noncoding regions remains more challenging than identifying variants in coding genes. This is in part due to sequencing and mapping artifacts, uncharacterized regulatory regions and hypermutation processes, inaccurate estimation of background mutation rates, and the challenge in understanding functional effects of noncoding mutations. The discovery of noncoding drivers from structural variants is further complicated by their sparsity compared to protein-coding genes, a paucity of obvious neutral events for constructing background models, and largely unexplored functional effects. Appropriate statistical methods that address these issues are needed to reliably identify noncoding drivers.

These caveats are epitomized in a recent analysis of noncoding somatic drivers in 2583 cancer whole genomes from 27 tumor types, which found that several significant noncoding elements, such as NEAT1 and MALAT1, harbored recurrent indels [93]. However, these mutations were not associated with changes in gene expression, high cancer cell fractions, or loss of heterozygosity. If the indels in these genes were due to an indirect mutational process rather than a selective one, they might exhibit distinct features. As the indels in NEAT1 and MALAT1 were strongly enriched in 2–5 bp-long events, these signatures suggest the variants are not driver events and are the result of a transcription-associated mutational process. Previously reported oncogenic effects of altered MALAT1 and NEAT1 expression may thus be unrelated to these indels.

In GBM, a recent study leveraged deep whole-genome sequencing of matched primary and recurrent tumors from 23 patients to infer the clonal evolution of recurrent genetic variants [71]. Driver mutations detectable in tumors of at least three patients were found in the coding regions of 28 genes, including those associated with early copy number changes, e.g., EGFR amplification and chromosome 7 gain, in 13 noncoding RNA genes, and in the *TERT* promoter region. The putative driver noncoding RNAs include SNHG14, KCNQ1OT1, TSIX, XIST, AC005154.6, AC108142.1, FZD10-AS1, HOTTIP, LINC00473, LINC00689, LINC00343, RP11-627G23.1, and RP3-399L15.3. The functional consequences of these variants, if any, have yet to be characterized in any context. It is entirely possible that these variants also arise from transcription-associated mutational processes, as seen with the recurrent indels in MALAT1 and NEAT1 in pancancer analyses. Still, the better described candidates from this list, such as SNHG14 and KCNQ1OT1, demonstrate expression changes that drive tumorigenic processes in GBM (Table S1). Whether these expression changes are a consequence of the genetic variants uncovered by the evolutionary trajectories of these patients requires further investigation.

The discovery of noncoding drivers in GBM will greatly benefit from technical improvements, including even sequence coverage, longer and accurate reads, and improved variant-calling methods. A continued effort for annotating functional noncoding elements will be crucial for increasing both the power to discover infrequently mutated driver elements and dissect their biological implications. This demonstrates a need for deeper investigation of noncoding alterations that are specifically relevant to GBM given the tissue- and disease-specific patterns that characterize both noncoding RNA expression and noncoding genome changes.

### 5.2. lncRNA Expression Deregulation

**Figure 3:**
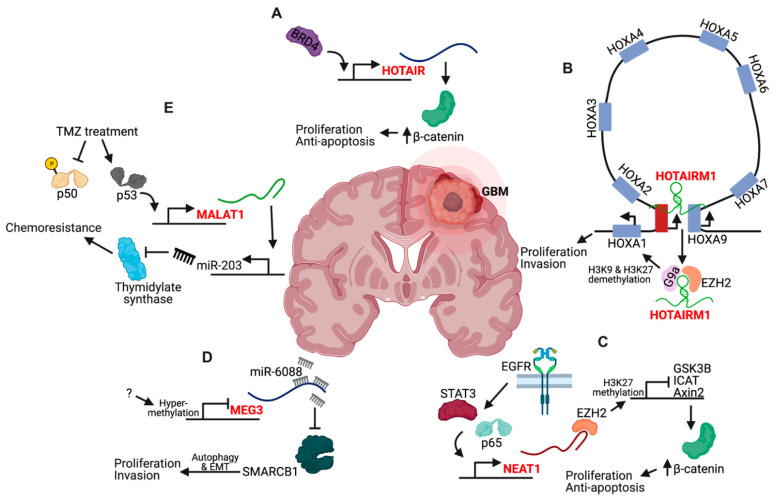
Mechanisms of lncRNAs in GBM. (**A**) The Bromodomain Containing 4 (BRD4) protein binds the *HOTAIR* promoter and regulates its expression for proliferative and antiapoptotic effects via β-catenin. (**B**) The HOTAIRM1 lncRNA is expressed within the HOXA gene cluster and promotes DNA looping by scaffolding with the HOXA9 gene to form a topologically associated domain. HOTAIRM1 also sequesters the G9a and EZH2 epigenetic modifiers, preventing methylation of the HOXA1 promoter and increasing its expression. (**C**) EGFR signaling stimulates the expression of NEAT1 in a STAT3- and p65-dependent manner. NEAT1 binds EZH2 and triggers the promoter methylation and decreased expression of GSK3B, ICAT, and Axin2, which normally sequester β-catenin in the cytoplasm to prevent transcriptional activity. (**D**) Hypermethylation of the MEG3 promoter results in decreased expression, which enables the accumulation of miR-6088 and inhibition of SMARCB1. This promotes autophagy and an epithelial-to-mesenchymal transition (EMT) program that drives proliferation and invasion. (**E**) Therapeutic intervention with TMZ triggers Ser329 phosphorylation of p50, which prevents its recruitment to the *MALAT1* promoter while promoting p53 recruitment. Upregulated MALAT1 drives miR-203 expression, which targets thymidylate synthase for degradation and contributes to therapeutic resistance.

Table S1 lists a survey of lncRNAs whose deregulated expression has been implicated in GBM. Interestingly, of the 107 candidates, only 21 have been examined in more than one publication (CASC2, XIST, HOTAIRM1, NEAT1, FGD5-AS1, DGCR5, SNHG7, HOXA-AS2, MEG3, HOTAIR, H19, DLEU1, MALAT1, AGAP2-AS1, SBF2-AS1, miR155HG, AHIF, LINC00152, LINC00470, SNHG14, KCNQ1OT1). Of these 21 candidates, only HOTAIR, HOTAIRM1, NEAT1, MEG3, and MALAT1 appear in more than two publications within the last three years with HOTAIR being the most well-studied overall, with 9 publications (Figure 3). The HOTAIR gene is located within the HOXC gene cluster and encodes a 2.2 kb lncRNA that is shuttled from chromosome 12 to chromosome 2 by the SUZ12 subunit of the Polycomb repressive complex 2 (PRC2) for silencing of the HOXD locus [94]. The 5′ end of HOTAIR interacts with PRC2, while the 3′ end interacts with the histone demethylase LSD1 [95]. In GBM, HOTAIR expression is upregulated via the Bromodomain and extraterminal domain protein BRD4, and induces β-catenin activity by an unknown mechanism for increased cellular proliferation, migration, and invasion (Figure 3A) [96]. HOTAIR is required for GBM tumorigenesis in vivo, thus increasing its potential as a therapeutic target [97]. More work is needed to investigate whether the roles of HOTAIR in histone methylation are operative in GBM.

HOTAIRM1 is located between the HOXA1 and HOXA2 genes and is normally expressed in cells of the myeloid lineage [98,99]. In GBM, HOTAIRM1 is upregulated and promotes proliferation, migration, and invasion of tumor cells via several mechanisms. HOTAIRM1 was found to sponge miR-153-5p, which directly targets SNAl2. This reinforces a positive feedback loop whereby SNAl2 upregulates the expression of HOTAIRM1 and suppresses negative regulation by CDH1 [100]. In vivo experiments demonstrated that HOTAIRM1 knockdown decreases tumor growth by regulating expression of the HOXA1 gene (Figure 3B). HOTAIRM1 mediated demethylation of histone H3K9 and H3K27 and reduced DNA methylation levels by sequestering epigenetic modifiers G9a and EZH2 away from the transcription start site of HOXA1 [101]. It was further demonstrated by Capture-C analysis that HOTAIRM1 facilitates DNA looping between its locus, which possesses enhancer function for transcriptional activation, and HOXA genes [102].

NEAT1 is a 3.2 kb nuclear lncRNA transcribed from the multiple endocrine neoplasia locus on chromosome 11 [103]. NEAT1 is upregulated during GBM tumorigenesis and TMZ resistance. NEAT1 levels are regulated by EGFR pathway activity in a STAT3- and NFκB-dependent fashion (Figure 3C) [104]. Moreover, NEAT1 is critical for GBM cell growth and invasion by increasing β-catenin nuclear transport and downregulating ICAT, GSK3B, and Axin via EZH2 binding to mediate H3K27 trimethylation at their promoters. It was later found that miR-370-3p is downregulated in GBM, which contributes to the increased expression of NEAT1 by reducing its inhibition [105]. The effect of NEAT1 on β-catenin activity also contributes to therapeutic resistance—TMZ induces the expression of the HMGB1 protein, which, via TLR2, increases NEAT1 expression [106].

MEG3 is an imprinted, maternally expressed lncRNA with at least 12 different isoforms generated by alternative splicing [107]. The downregulation of MEG3 in GBM cells causes increased proliferation, migration, and expression of epithelial-to-mesenchymal transition (EMT) genes by sponging miR-6088, which targets SMARCB1 (Figure 3D) [108]. The mechanism of MEG3 in promoting the EMT phenotype is in part due to autophagy since MEG3-induced EMT could be partially reversed by autophagy inhibitors in GBM cells [109]. More recently, it was found that MEG3 loss occurs in GBM stem cells through epigenetic silencing of the *DLK1-DIO3* region where it is located [110].

MALAT1 is a large, infrequently spliced lncRNA, which is highly conserved amongst mammals and highly expressed in the nucleus [103]. In GBM, MALAT1 is also implicated in both tumorigenesis and recurrence, and is currently under investigation for its ability to promote the resistance of GBM cells to current therapeutic agents. During tumorigenesis, MALAT1 upregulation promotes increased proliferation and tumor formation in vivo by promoting ZHX1 expression via a competitive, endogenous mechanism that sponges miR-199a [111]. In the context of therapy, MALAT1 promotes resistance by upregulating miR-203, which targets thymidylate synthase (Figure 3E) [112]. More recently, it was found that MALAT1 expression was coregulated by p50 and p53 via novel NFκB and p53-binding sites in the proximal MALAT1 coding region [113]. TMZ treatment triggers the phosphorylation of p50, which inhibits its recruitment while concomitantly increasing p53 recruitment. Delivery of nanoparticle-encapsulated anti-MALAT1 siRNA in vivo increased the efficacy of TMZ treatment in mice bearing intracranial GBM xenografts, suggesting that reducing MALAT1 sensitizes patient-derived GBM cells to therapy [114].

Interestingly, a common mechanism among all of these well-described lncRNAs in GBM is their ability to sponge and thereby regulate miRNA targeting and the inhibition of mRNAs. Increasingly, it is also becoming apparent that specific ceRNA networks between lncRNAs, miRNAs, and mRNAs or proteins may provide prognostic information for GBM patients, with potential therapeutic implications. ceRNA networks between miRNAs and mRNAs were first described in the context of the somatic genomic landscape of GBM, where 133 miR:mRNA associations defined a putative miR regulatory network that was associated with GBM signature genes PDGFRA, EGFR, NF1, and PTEN, and the bulk GBM transcriptional subtypes [70]. Since then, interest in these ceRNA networks has skyrocketed, with over 80 studies published on ceRNA networks in GBM, and 60 of these published in the last two years. For example, the interaction between 2 lncRNAs (NORAD, XIST), 5 miRNAs (hsa-miR-3613, has-miR-371, has-miR-32, has-miR-92) and 2 mRNAs (LYZ, PIK3AP1) may affect immune and tumor microenvironment variations and act as a prognostic biomarker in GBM [115]. Comprehensive analyses of these networks have yielded intricate maps of coexpression variation in RNA species that affect up to 60 mRNAs. Although these intricate networks have yet to inform GBM biology, they have demonstrated utility in stratifying patient survival, thus serving as potential prognostic biomarkers [116,117]. Utilizing these networks to inform GBM biology and better understand the malignant transformation of tumor cells may benefit from leveraging atlases of neurodevelopmental gene expression programs in relevant cell types, particularly radial glia and intermediate progenitors [118]. Although some cell type-specific neurodevelopmental lncRNAs have been implicated in GBM (Table 1), more work is needed to determine whether additional developmental signatures are relevant in GBM. Given the region-specific expression patterns of lncRNAs in the adult brain, whether noncoding changes during tumorigenesis in different brain regions retain their spatially distinct expression, demonstrate plasticity towards a common primitive development like program, or both remains unknown.

It is also prudent to recognize that lncRNAs whose expression levels do not change in GBM compared to normal brain cell populations may also be relevant for tumor evolution. For instance, the basal expression of these species may be co-opted by tumorigenic transcriptional processes that render them a necessary component for continued malignant transformation. A more comprehensive understanding of the noncoding transcriptome dysregulation in GBM may also enable better liquid biopsy-based detection of GBM tumors. The characterization of the extracellular RNA landscape associated with cancer stem cells highlights this diagnostic potential for detection of cancer-associated transcripts using cerebrospinal fluid, blood, or urine [10]. Furthermore, improved knowledge of the noncoding transcriptome in GBM may lead to more effective utilization of RNA-based therapeutics.

## 6. RNA-Based Therapeutics

The current development and implementation of SARS-CoV2 mRNA vaccines demonstrates one example of the groundbreaking advances in modern medicine achieved from the use of RNA-based therapeutics. In the context of ncRNAs, tissue-specific expression is a feature that strongly advocates for these molecules as potential targets of therapy, as well as disease biomarkers. A CRISPRi noncoding library that targeted 16,401 lncRNA genes in seven human cell lines, including six transformed cell lines and induced pluripotent stem cells, demonstrated that a remarkable 89% of lncRNA gene hits modified growth in just one of the cell lines tested with no hits common to all seven cell lines [119]. The screen revealed 65 lncRNA hits that inhibit growth of the GBM cell line U87 with top hits including LINC00263, XLOC_14806, LINC00909, MIR29A, CTB-51J22.1, RP11-416I2.1, and PVT1. Although this greatly decreases the probability for the existence of a master regulatory oncogenic lncRNA, it highlights the potential specificity of lncRNA-based therapeutics along with a possible decreased likelihood of off-target effects. For example, a recent CRISPRi screen of 5689 lncRNA loci in human GBM cells in the context of radiation therapy identified lncRNA Glioma Radiation Sensitizer 1, lncGRS-1, a primate-conserved, nuclear-enriched noncoding transcript whose knockdown inhibited the growth and proliferation of primary adult and pediatric glioma cells, but not the viability of normal brain cells. Antisense oligonucleotides directed against lncGRS-1 selectively decreased tumor growth and sensitized GBM cells to radiation therapy [120].

NcRNAs can also be therapeutically targeted using gene therapy, such as an RNA interference (RNAi) approach. It was previously thought that RNA was inappropriate as a molecular therapeutic due to its instability. The physiochemical properties of RNA reduces its bioavailability in the body and ability to act in target cells. It is a negatively charged, hydrophilic molecule, preventing it from crossing cell membranes by passive means. Additionally, once RNA has entered the cell, nucleases within the cytoplasm will destroy exogenous, naked RNA strands as an evolutionary defense against viral infection. Furthermore, the presence of extracellular RNA may trigger an immune response, making its therapeutic use limited by potential harm to GBM patients. Recent advances in RNAi therapeutics have involved both the chemical stabilization of synthetic RNAs, as well as the development of nanodelivery systems for successful introduction into target cells [121]. Such developments have promoted RNAs as next-generation personalized therapeutics for a spectrum of diseases, including GBM. Synthetic RNAs are easy and inexpensive to produce, allowing therapeutics to be developed rapidly and approved more quickly than chemotherapy drugs. Once general safety, efficacy, effective delivery systems, and acceptability are established, the ideal RNAi delivery system must be biocompatible, biodegradable, nonimmunogenic, and nontoxic, to minimize or avoid harmful side effects when used clinically [122].

Encapsulating RNA molecules in nanodelivery vehicles avoids or blocks the immune recognition of RNA molecules and improves delivery to target tissues [123]. Both viral and nonviral vectors have been reported as RNAi therapeutic nanodelivery vehicles, with viral vectors showing superiority in delivery and interference. However, nonviral vectors possess unique advantages such as a lower immune response, decreased renal and phagocytic clearance, and a favorable safety profile [124]. Nonviral systems are positively charged and include lipoplexes, polyplexes, micelleplexes, and inorganic materials, such as gold, silver, platinum, and iron oxide, that electrostatically complex with the nucleic acids and improve delivery. Furthermore, multifunctionality can be imparted to these carrier systems so that they can effectively carry the nucleic acids to the targeted site while protecting their integrity during the transport.

A few preclinical studies of RNAi therapeutics have shown promising results in the treatment of GBM. As mentioned previously, intracranial injection of nanoparticle-encapsulated anti-MALAT1 increased the efficacy of TMZ in orthotopic xenograft models of GBM [114]. A number of other prominent preclinical examples of RNAi successes in vivo in GBM include: (1) intravenous administration of liposome-encapsulated siRNA targeting PLK1; (2) intravenous administration of a miR-7 mimic entrapped in an integrin targeted polymeric nanoparticle; (3) intratumoral administration of exosomes containing miR-146b rapidly reduced tumor volume in a rat primary brain tumor model [125,126,127]. This highlights the potential for not only inhibiting oncogenic molecules as a therapeutic strategy, but also reintroducing tumor suppressors that were inactivated during tumor evolution. Spherical nucleic acids (SNAs) are gold nanoparticle cores covalently conjugated with a corona of densely packed oligonucleotides. Intravenous administration of gold SNAs conjugating with Bcl2L12-targeting siRNAs reduced Bcl2L12 mRNA levels, increased apoptosis, and decreased tumor burden in a mouse orthotopic xenograft model [128]. This led to an early phase I clinical trial to test the safety of the Bcl2L12-targeting SNA, NU-0129, in recurrent GBM or gliosarcoma patients undergoing surgery (NCT03020017). Finally, intravenous administration of gold SNAs with conjugated miR-182 duplexes reduced tumor size and increased overall survival in preclinical studies, which increases potential for expansion to early clinical trials [129]. Thus, the use of RNA therapeutics is an exciting opportunity to improve the treatment of GBM tumors but its true potential can only be realized with more comprehensive identification and biological understanding of the noncoding molecular drivers of tumorigenesis and recurrence.

## 7. Conclusions and Perspectives

We are now beginning to appreciate that ncRNAs of various sizes facilitate a diverse range of molecular and cellular functions in both the healthy and disease contexts. To date, a broad categorization of lncRNAs as guides, scaffolds, sponges, or peptides, accurately describes the functions and binding patterns of a large majority of lncRNA species. Some lncRNAs may even possess different functions in a context-dependent fashion, and as we learn more about the structural determinants of these mechanisms, it will be necessary to update the current classification scheme for accurate and comprehensive characterization. We are also beginning to understand how lncRNA expression patterns are generally tissue-specific and likely represent unexplored mechanisms for disease processes. This is particularly true in the brain, which demonstrates the largest repertoire of lncRNA transcripts in the body and may parallel the complex cell fate specification programs in the nervous system.

The vast array of ncRNAs in the nervous system may provide ample raw material for malignant cells to rapidly sample and hijack for the increased selective advantages and adaptability that support the deadly brain cancer GBM. As we learn more about the molecular mechanisms used by cancer cells for driving malignant processes, such as the propagation of extrachromosomal oncogenes or the population dynamics of early, founding genetic clones, continued investigation will be necessary for determining whether dysregulated DNA and protein elements require RNA components. Many studies have begun describing how the deregulated expression of specific lncRNAs contribute to cellular phenotypes related to tumorigenesis and therapeutic resistance. However, more work is needed to understand whether mutations in lncRNA genes may represent driver events that are necessary for tumorigenesis or recurrence. There should also be consideration of lncRNA dependencies in GBM that are not defined by genetic or expression anomalies. Targeting ncRNAs necessary for integral tumor cell processes may circumvent the limitations inherent to directly targeting master regulatory proteins given their important roles in normal stem cell populations versus the context-specific usage of the RNA elements. As new technologies are continually developed and our understanding of GBM improves, it becomes more feasible to leverage various classes of therapeutic molecules and the potential for a significant improvement in the clinical management of this disease becomes more realistic.

## Figures and Tables

**Table 1:** Signatures of neurodevelopmental cell types. (Bold indicates lncRNAs implicated in GBM).

Cell Type	lncRNA Signature	mRNA Signature	Functional Ontologies
Endothelia	LINC-MILR1-3, SLC38A3, **LINC00152**, RP11-401P9.4, **MIR4435-HG**, **LINC00339**, AP000459.4, AC127904.2, RP11-161M6.2, RP11-417F21.1, TRIM52-AS1, CTD-2081C10.7, RP11-296I10.3, RP11-532M24.1	GPR116, ITM2A, C1orf54, GNG11, COL4A1, ECSCR, EMCN, LAMA4, ECM1, RAPGEF4, A2M, IGFBP7, CD93, FLT1, RNF144B	Angiogenesis Regulation of vasculature development Hemostasis Response to oxygen levels Blood coagulation Coagulation Regulation of angiogenesis Response to decreased oxygen levels Response to hypoxia Extracellular matrix organization
Radial Glia	Z83001.1, RP11-731J8.2, LINC00943, RP3-418C23.2, RP11-1002K11.1, **MAGI2-AS3**, RP11-421L21.3, LINC-FZD3-3, LINC-FZD8-1, **LINC00263**, **EIF3J-AS1**, **LOC646329**, LINC-KREMEN1-1, RUSC1-AS1, DGKK	GPX3, ATP1A2, BCAN, MOXD1, LIPG, CLU, FAM107A, ANXA2, VIM, GFAP, PPAP2B, ZFP36L1, GATM, TNC, HES1	Negative regulation of nervous system development Negative regulation of neuron development Negative regulation of neurogenesis Glial cell differentiation Response to mechanical stimulus Regulation of neuron differentiation Extracellular matrix organization Extracellular structure organization Positive regulation of neuroblast proliferation
Dividing Radial Glia	**UHRF1**, CTB-175P5.4, RP11-138A9.1, RP11-143K11.1, AC004447.2, SNORA59B, CTC-503J8.6, RP11-138A9.2, RP11-95D17.1, THAP9-AS1, **SNHG1**, CTD-2017D11.1, RP11-58B17.2, DYNLL1-AS1	MKI67, KIF15, CCNB2, CDK1, UBE2C, FAM64A, NDC80, AURKB, MELK, TPX2, CDCA5, HIST1H1B, BIRC5, ZWINT, TOP2A	Mitotic cell cycle Nuclear division Organelle fission Mitotic nuclear division Chromosome segregation Regulation of cell cycle process Cell cycle checkpoint Chromosome organization DNA repair Microtubule-based process
Intermediate Progenitor	LINC-TMEM200C-1, RP11-798G7.8, RP11-35IJ23.1-AS1, RP3-326L13.3, CTD-2245E15.3, C1orf132, AC084018.1, RP11-73O6.3, RP11-594N15.3, RP11-436D23.1, AC0838848.8, DGCR11, RP11-456K23.1, RP6-24A23.3, RP1-20C7.6	PPP1R17, EOMES, NHLH1, SSTR2, SETD7, CCDC129, SIPA1L2, NPR3, FAM60A, SLCO4C1, TRIM45, INHBB, UBL7, STX8, TMEM206	Dicarboxylic acid biosynthetic process Glutamine family amino acid biosynthetic process GPI anchor metabolic process Regulation of triglyceride biosynthetic process Glutamate metabolic process Neuroblast proliferation Neuromuscular synaptic transmission GPI anchor biosynthetic process Positive regulation of triglyceride metabolic process Positive regulation of triglyceride biosynthetic process
New Neuron	RP5-1024G6.8, LINC-PTCHD2-3, RP11-513M16.8, RP11-661O13.1, RP11-524C21.2, RP11-356K23.1, LINC01105	SLC24A2, NRP1, RASGEF1, PALMD, SEMA3C, KCNQ3, UNC5D, SLC17A6, DOK6, SEZ6, DCC, SORBS2, FAM126A, ZNF804A, PPP2R2B	Limb bud formation Cardiac ventricle morphogenesis Cardiac chamber morphogenesis Axon extension Regulation of neuron differentiation Neuron projection extension Positive regulation of neuron differentiation Positive regulation of neurogenesis Glial cell development Regulation of neuron projection development
Maturing Neuron	MIR137HG, **LINC00599**, PWAR6, SIK3-IT1, RP11-53O19.3, RP11-402L6.1, RP11-18I14.10, RP11-486F17.1, NAV2-AS3, DAPK1-IT1, RP11-397O4.1, RP11-64K12.10, LINC00643, RP3-462E2.5, LINC-TMEM182-5	SLC44A5, GRIN2B, CCBE1, CDH13, CAMK2B, SATB2, ARPP21, ADRA2A, DAB1, GLRA2, GPR85, KIAA0319, MCTP1, ADCY1, FLRT2	Limb bud formation Cardiac ventricle morphogenesis Cardiac chamber morphogenesis Axon extension Regulation of neuron differentiation Neuron projection extension Positive regulation of neuron differentiation Positive regulation of neurogenesis Glial cell development Regulation of neuron projection development
Interneuron	DLX-AS1, RP11-588P7.1, **SOX2-OT**, GS1-18A18.1, **MEG3**, LINC-DKFZP761K2322-2, GRIP2, AC87393.1, LINC00966, RP11-450H6.3, RP13-514E23.1, RP11-379H18.1, RP11-69E11.4, AC012358.8, LINC-TBCC-1	ERBB4, GAD1, MAF, DLX2, NRXN3, FAM65B, DLX5, PLS3, PDZRN3, LHX6, DLX6, THRB, SCGN, IGF1, CELF4	GABA synthesis, release, reuptake and degradation Transmission across chemical synapses Neurotransmitter release cycle Nuclear receptor transcription pathway Signaling by ERBB2 Signaling by FGFR Signaling by FGFR in disease Neuronal system Downstream signal transduction Downstream signaling of activated FGFR

## Data Availability

Not applicable.

## Supplementary Materials

The following are available online at https://www.mdpi.com/2072-6694/13/7/1604/s1, Table S1: A survey of lncRNAs whose deregulated expression changes have been implicated in GBM.
