# Supplementary material for: Long, Noncoding RNA Dysregulation in Glioblastoma
Source: Cancers (Basel). 2021 Mar 31;13(7):1604. doi: 10.3390/cancers13071604 (PMC8037018; doi:10.3390/cancers13071604)
Supplement: Supplementary file 1 [file cancers-13-01604-s001.pdf]

Supplementary Materials

Long, Noncoding RNA Dysregulation in Glioblastoma

Patrick A. DeSouza, Xuan Qu, Hao Chen, Bhuvic Patel, Christopher A. Maher and Albert H. Kim

Table S1. A survey of lncRNAs whose deregulated expression changes have been implicated in GBM. *PubMed search terms: ("glioblastoma" OR "gbm") AND ("long noncoding RNA" OR "lncRNA").*

lncRNA	Expression in GBM	References
AC016405.3	Downregulated	Ren S, Xu Y. AC016405.3, a novel long noncoding RNA, acts as a tumor suppressor through modulation of TET2 by microRNA-19a-5p sponging in glioblastoma. Cancer Sci. 2019 May;110(5):1621-1632. doi: 10.1111/cas.14002. Epub 2019 Apr 23. PMID: 30888082; PMCID: PMC6500966.
AC068643.1	Downregulated	Huang GH, Pei YC, Yang L, Mou KJ, Tang JH, Xiang Y, Liu J, Lv SQ. Integrative transcriptome analysis identified a BMP signaling pathway-regulated lncRNA AC068643.1 in IDH mutant and wild-type glioblastomas. Oncol Lett. 2020 Jul;20(1):75-84. doi: 10.3892/ol.2020.11542. Epub 2020 Apr 15. PMID: 32565936; PMCID: PMC7285920.
ADAMTS9-AS2	Upregulated	Yan Y, Xu Z, Chen X, Wang X, Zeng S, Zhao Z, Qian L, Li Z, Wei J, Huo L, Li X, Gong Z, Sun L. Novel Function of lncRNA ADAMTS9-AS2 in Promoting Temozolomide Resistance in Glioblastoma via Upregulating the FUS/MDM2 Ubiquitination Axis. Front Cell Dev Biol. 2019 Oct 2;7:217. doi: 10.3389/fcell.2019.00217. PMID: 31632968; PMCID: PMC6783494.
AGAP2-AS1	Upregulated	Luo W, Li X, Song Z, Zhu X, Zhao S. Long non-coding RNA AGAP2-AS1 exerts oncogenic properties in glioblastoma by epigenetically silencing TFPI2 through EZH2 and LSD1. Aging (Albany NY). 2019 Jun 11;11(11):3811-3823. doi: 10.18632/aging.102018. PMID: 31186379; PMCID: PMC6594811.<br>Tian Y, Zheng Y, Dong X. AGAP2-AS1 serves as an oncogenic lncRNA and prognostic biomarker in glioblastoma multiforme. J Cell Biochem. 2019 Jun;120(6):9056-9062. doi: 10.1002/jcb.28180. Epub 2018 Dec 7. PMID: 30525219.
AHIF	Upregulated	Liao K, Ma X, Chen B, Lu X, Hu Y, Lin Y, Huang R, Qiu Y. Upregulated AHIF-mediated radioresistance in glioblastoma. Biochem Biophys Res Commun. 2019 Feb 5;509(2):617-623. doi: 10.1016/j.bbrc.2018.12.136. Epub 2018 Dec 31. PMID: 30606477.<br>Dai X, Liao K, Zhuang Z, Chen B, Zhou Z, Zhou S, Lin G, Zhang F, Lin Y, Miao Y, Li Z, Huang R, Qiu Y, Lin R. AHIF promotes glioblastoma progression and radioresistance via exosomes. Int J Oncol. 2019 Jan;54(1):261-270. doi: 10.3892/ijo.2018.4621. Epub 2018 Nov 2. PMID: 30387845.
BCAR4	Upregulated	Wang Z, Wang L, Liang Z, Xi Y. Long Non-Coding RNA BCAR4 Promotes Growth, Invasion and Tumorigenicity by Targeting miR-2276 to Upregulate MMP7 Expression in Glioma. Onco Targets Ther. 2019 Dec 12;12:10963-10973. doi: 10.2147/OTT.S226026. PMID: 31849498; PMCID: PMC6913310.
BDNF-AS	Downregulated	Lv X, Gu C, Guo S. Activation of BDNF-AS/ADAR/p53 Positive Feedback Loop Inhibits Glioblastoma Cell Proliferation. Neurochem Res. 2020 Feb;45(2):508-518. doi: 10.1007/s11064-019-02943-w. Epub 2020 Jan 14. PMID: 31939089.

CASC2	Downregulated	Skiriute D, Stakaitis R, Steponaitis G, Tamasauskas A, Vaitkiene P. The Role of CASC2 and miR-21 Interplay in Glioma Malignancy and Patient Outcome. Int J Mol Sci. 2020 Oct 27;21(21):7962. doi: 10.3390/ijms21217962. PMID: 33120918; PMCID: PMC7663706.<br>Wang J, Qin C, Zhong C, Wen Y, Ke S, Liao BO. Long non-coding RNA CASC2 targeting miR-18a suppresses glioblastoma cell growth, metastasis and EMT in vitro and in vivo. J Biosci. 2020;45:107. PMID: 32975234.
CASP5	Upregulated	Zhou Y, Dai W, Wang H, Pan H, Wang Q. Long non-coding RNA CASP5 promotes the malignant phenotypes of human glioblastoma multiforme. Biochem Biophys Res Commun. 2018 Jun 12;500(4):966-972. doi: 10.1016/j.bbrc.2018.04.217. Epub 2018 May 2. PMID: 29715460.
CDR1as	Downregulated	Lou J, Hao Y, Lin K, Lyu Y, Chen M, Wang H, Zou D, Jiang X, Wang R, Jin D, Lam EW, Shao S, Liu Q, Yan J, Wang X, Chen P, Zhang B, Jin B. Circular RNA CDR1as disrupts the p53/MDM2 complex to inhibit Gliomagenesis. Mol Cancer. 2020 Sep 7;19(1):138. doi: 10.1186/s12943-020-01253-y. PMID: 32894144; PMCID: PMC7487905.
CRNDE	Upregulated	Kiang KMY, Leung GKK. Clinical significance of CRNDE transcript variants in glioblastoma multiforme. Noncoding RNA Res. 2017 Jul 26;2(2):119-121. doi: 10.1016/j.ncrna.2017.07.001. PMID: 30159429; PMCID: PMC6096424.
DCST1-AS1	Upregulated	Hu S, Yao Y, Hu X, Zhu Y. LncRNA DCST1-AS1 downregulates miR-29b through methylation in glioblastoma (GBM) to promote cancer cell proliferation. Clin Transl Oncol. 2020 Dec;22(12):2230-2235. doi: 10.1007/s12094-020-02363-1. Epub 2020 May 16. PMID: 32418155.
DGCR5	Downregulated	Wu X, Hou P, Qiu Y, Wang Q, Lu X. Large-Scale Analysis Reveals the Specific Clinical and Immune Features of DGCR5 in Glioma. Onco Targets Ther. 2020 Jul 31;13:7531-7543. doi: 10.2147/OTT.S257050. PMID: 32801772; PMCID: PMC7402863.<br>Yang F, Huang YL. DGCR5 suppresses the EMT of pediatric primary glioblastoma multiforme cell and serves as a prognostic biomarker. Eur Rev Med Pharmacol Sci. 2019 Nov;23(22):10024-10034. doi: 10.26355/eurrev_201911_19569. PMID: 31799672.
DLEU1	Upregulated	Liu X, Chen R, Liu L. SP1-DLEU1-miR-4429 feedback loop promotes cell proliferative and anti-apoptotic abilities in human glioblastoma. Biosci Rep. 2019 Dec 20;39(12):BSR20190994. doi: 10.1042/BSR20190994. PMID: 31713587; PMCID: PMC6900472.<br>Wang J, Quan X, Peng D, Hu G. Long non-coding RNA DLEU1 promotes cell proliferation of glioblastoma multiforme. Mol Med Rep. 2019 Aug;20(2):1873-1882. doi: 10.3892/mmr.2019.10428. Epub 2019 Jun 26. PMID: 31257517.
EGFR-AS1	Upregulated	Dong ZQ, Guo ZY, Xie J. The lncRNA EGFR-AS1 is linked to migration, invasion and apoptosis in glioma cells by targeting miR-133b/RACK1. Biomed Pharmacother. 2019 Oct;118:109292. doi: 10.1016/j.biopha.2019.109292. Epub 2019 Aug 20. PMID: 31545240.
FAM225B	Upregulated	Li J, Zhang Q, Ge P, Zeng C, Lin F, Wang W, Zhao J. *FAM225B* Is a Prognostic lncRNA for Patients with Recurrent Glioblastoma. Dis Markers. 2020 Nov 22;2020:8888085. doi: 10.1155/2020/8888085. PMID: 33299501; PMCID: PMC7704151.
FEZF1-AS1	Upregulated	Luo L, Zhang Y, He H, Chen C, Zhang B, Cai M. LncRNA FEZF1-AS1 Sponges miR-34a to Upregulate Notch-1 in Glioblastoma. Cancer Manag Res. 2020 Mar 11;12:1827-1833. doi: 10.2147/CMAR.S240531. PMID: 32210625; PMCID: PMC7075242.

FGD5-AS1	Upregulated	Su D, Ji Z, Xue P, Guo S, Jia Q, Sun H. Long-Noncoding RNA FGD5-AS1 Enhances the Viability, Migration, and Invasion of Glioblastoma Cells by Regulating the miR-103a-3p/TPD52 Axis. Cancer Manag Res. 2020 Jul 27;12:6317-6329. doi: 10.2147/CMAR.S253467. PMID: 32848452; PMCID: PMC7425657.<br>Wu L, Zhu X, Song Z, Guo M, Liang J, Yan D. FGD5-AS1 facilitates glioblastoma progression by activation of Wnt/β-catenin signaling via regulating miR-129-5p/HNRNPK axis. Life Sci. 2020 Sep 1;256:117998. doi: 10.1016/j.lfs.2020.117998. Epub 2020 Jun 22. PMID: 32585241.
FLVCR1-AS1	Upregulated	Gao W, Li H, Liu Y, Zhang Y, Zhao H, Liu F. Long non-coding RNA FLVCR1-AS1 promotes glioma cell proliferation and invasion by negatively regulating miR-30b-3p. Mol Med Rep. 2020 Aug;22(2):723-732. doi: 10.3892/mmr.2020.11149. Epub 2020 May 15. PMID: 32626942; PMCID: PMC7339652.
GAPLINC	Upregulated	Chen HH, Zong J, Wang SJ. LncRNA GAPLINC promotes the growth and metastasis of glioblastoma by sponging miR-331-3p. Eur Rev Med Pharmacol Sci. 2019 Jan;23(1):262-270. doi: 10.26355/eurrev_201901_16772. PMID: 30657584.
GAS5	Downregulated	Wang Y, Xin S, Zhang K, Shi R, Bao X. Low GAS5 Levels as a Predictor of Poor Survival in Patients with Lower-Grade Gliomas. J Oncol. 2019 Feb 3;2019:1785042. doi: 10.1155/2019/1785042. PMID: 30853980; PMCID: PMC6377997.
H19	Upregulated	Xiao Y, Zhu Z, Li J, Yao J, Jiang H, Ran R, Li X, Li Z. Expression and prognostic value of long non-coding RNA H19 in glioma via integrated bioinformatics analyses. Aging (Albany NY). 2020 Feb 20;12(4):3407-3430. doi: 10.18632/aging.102819. Epub 2020 Feb 20. PMID: 32081833; PMCID: PMC7066912.<br>Li W, Jiang P, Sun X, Xu S, Ma X, Zhan R. Suppressing H19 Modulates Tumorigenicity and Stemness in U251 and U87MG Glioma Cells. Cell Mol Neurobiol. 2016 Nov;36(8):1219-1227. doi: 10.1007/s10571-015-0320-5. Epub 2016 Mar 16. PMID: 26983719; PMCID: PMC5047947.<br>Jiang X, Yan Y, Hu M, Chen X, Wang Y, Dai Y, Wu D, Wang Y, Zhuang Z, Xia H. Increased level of H19 long noncoding RNA promotes invasion, angiogenesis, and stemness of glioblastoma cells. J Neurosurg. 2016 Jan;124(1):129-36. doi: 10.3171/2014.12.JNS1426. Epub 2015 Aug 14. PMID: 26274999.<br>Wu W, Hu Q, Nie E, Yu T, Wu Y, Zhi T, Jiang K, Shen F, Wang Y, Zhang J, You Y. Hypoxia induces H19 expression through direct and indirect Hif-1*α* activity, promoting oncogenic effects in glioblastoma. Sci Rep. 2017 Mar 22;7:45029. doi: 10.1038/srep45029. PMID: 28327666; PMCID: PMC5361208.
HAS2-AS1	Upregulated	Zhang L, Wang H, Xu M, Chen F, Li W, Hu H, Yuan Q, Su Y, Liu X, Wuri J, Yan T. Long noncoding RNA HAS2-AS1 promotes tumor progression in glioblastoma via functioning as a competing endogenous RNA. J Cell Biochem. 2020 Jan;121(1):661-671. doi: 10.1002/jcb.29313. Epub 2019 Aug 5. PMID: 31385362.
HIF1A-AS2	Upregulated	Mineo M, Ricklefs F, Rooj AK, Lyons SM, Ivanov P, Ansari KI, Nakano I, Chiocca EA, Godlewski J, Bronisz A. The Long Non-coding RNA HIF1A-AS2 Facilitates the Maintenance of Mesenchymal Glioblastoma Stem-like Cells in Hypoxic Niches. Cell Rep. 2016 Jun 14;15(11):2500-9. doi: 10.1016/j.celrep.2016.05.018. Epub 2016 Jun 2. PMID: 27264189; PMCID: PMC4909547.

HMMR-AS1	Upregulated	Li J, Ji X, Wang H. Targeting Long Noncoding RNA HMMR-AS1 Suppresses and Radiosensitizes Glioblastoma. Neoplasia. 2018 May;20(5):456-466. doi: 10.1016/j.neo.2018.02.010. Epub 2018 Mar 22. PMID: 29574252; PMCID: PMC5915996.
HOTAIR	Upregulated	Yuan Z, Yang Z, Li W, Wu A, Su Z, Jiang B. Exosome-Mediated Transfer of Long Noncoding RNA HOTAIR Regulates Temozolomide Resistance by miR-519a-3p/RRM1 Axis in Glioblastoma. Cancer Biother Radiopharm. 2020 Jul 24. doi: 10.1089/cbr.2019.3499. Epub ahead of print. PMID: 32721218.<br>Zhang J, Chen G, Gao Y, Liang H. HOTAIR/miR-125 axis-mediated Hexokinase 2 expression promotes chemoresistance in human glioblastoma. J Cell Mol Med. 2020 May;24(10):5707-5717. doi: 10.1111/jcmm.15233. Epub 2020 Apr 12. PMID: 32279420; PMCID: PMC7214183.<br>Xavier-Magalhães A, Gonçalves CS, Fogli A, Lourenço T, Pojo M, Pereira B, Rocha M, Lopes MC, Crespo I, Rebelo O, Tão H, Lima J, Moreira R, Pinto AA, Jones C, Reis RM, Costello JF, Arnaud P, Sousa N, Costa BM. The long non-coding RNA HOTAIR is transcriptionally activated by HOXA9 and is an independent prognostic marker in patients with malignant glioma. Oncotarget. 2018 Feb 28;9(21):15740-15756. doi: 10.18632/oncotarget.24597. PMID: 29644006; PMCID: PMC5884661.<br>Tan SK, Pastori C, Penas C, Komotar RJ, Ivan ME, Wahlestedt C, Ayad NG. Serum long noncoding RNA HOTAIR as a novel diagnostic and prognostic biomarker in glioblastoma multiforme. Mol Cancer. 2018 Mar 20;17(1):74. doi: 10.1186/s12943-018-0822-0. PMID: 29558959; PMCID: PMC5861620.<br>Li H, Guan C. HOTAIR inhibits the proliferation of glioblastoma cells by targeting miR-219. Cancer Biomark. 2020;28(1):41-47. doi: 10.3233/CBM-190467. PMID: 32176624.<br>Huang K, Sun J, Yang C, Wang Y, Zhou B, Kang C, Han L, Wang Q. HOTAIR upregulates an 18-gene cell cycle-related mRNA network in glioma. Int J Oncol. 2017 Mar 7. doi: 10.3892/ijo.2017.3901. Epub ahead of print. PMID: 28350082.<br>Fang K, Liu P, Dong S, Guo Y, Cui X, Zhu X, Li X, Jiang L, Liu T, Wu Y. Magnetofection based on superparamagnetic iron oxide nanoparticle-mediated low lncRNA HOTAIR expression decreases the proliferation and invasion of glioma stem cells. Int J Oncol. 2016 Aug;49(2):509-18. doi: 10.3892/ijo.2016.3571. Epub 2016 Jun 8. PMID: 27277755; PMCID: PMC4922836.<br>Pastori C, Kapranov P, Penas C, Peschansky V, Volmar CH, Sarkaria JN, Bregy A, Komotar R, St Laurent G, Ayad NG, Wahlestedt C. The Bromodomain protein BRD4 controls HOTAIR, a long noncoding RNA essential for glioblastoma proliferation. Proc Natl Acad Sci U S A. 2015 Jul 7;112(27):8326-31. doi: 10.1073/pnas.1424220112. Epub 2015 Jun 25. PMID: 26111795; PMCID: PMC4500283.<br>Zhou X, Ren Y, Zhang J, Zhang C, Zhang K, Han L, Kong L, Wei J, Chen L, Yang J, Wang Q, Zhang J, Yang Y, Jiang T, Li M, Kang C. HOTAIR is a therapeutic target in glioblastoma. Oncotarget. 2015 Apr 10;6(10):8353-65. doi: 10.18632/oncotarget.3229. PMID: 25823657; PMCID: PMC4480757.
HOTAIRM1	Upregulated	Xie P, Li X, Chen R, Liu Y, Liu D, Liu W, Cui G, Xu J. Upregulation of HOTAIRM1 increases migration and invasion by glioblastoma cells. Aging (Albany NY). 2020 Dec 11;13(2):2348-2364. doi: 10.18632/aging.202263. Epub 2020 Dec 11. PMID: 33323548.

		Li Q, Dong C, Cui J, Wang Y, Hong X. Over-expressed lncRNA HOTAIRM1 promotes tumor growth and invasion through up-regulating HOXA1 and sequestering G9a/EZH2/Dnmts away from the HOXA1 gene in glioblastoma multiforme. J Exp Clin Cancer Res. 2018 Oct 30;37(1):265. doi: 10.1186/s13046-018-0941-x. PMID: 30376874; PMCID: PMC6208043. Shi T, Guo D, Xu H, Su G, Chen J, Zhao Z, Shi J, Wedemeyer M, Attenello F, Zhang L, Lu W. HOTAIRM1, an enhancer lncRNA, promotes glioma proliferation by regulating long-range chromatin interactions within HOXA cluster genes. Mol Biol Rep. 2020 Apr;47(4):2723-2733. doi: 10.1007/s11033-020-05371-0. Epub 2020 Mar 16. PMID: 32180085.
HOX-AS3	Upregulated	Wang X, Sun Y, Xu T, Qian K, Huang B, Zhang K, Song Z, Qian T, Shi J, Li L. HOXB13 promotes proliferation, migration, and invasion of glioblastoma through transcriptional upregulation of lncRNA HOXC-AS3. J Cell Biochem. 2019 Sep;120(9):15527-15537. doi: 10.1002/jcb.28819. Epub 2019 May 6. PMID: 31062400.
HOXA-AS2	Upregulated	Shou J, Gao H, Cheng S, Wang B, Guan H. LncRNA HOXA-AS2 promotes glioblastoma carcinogenesis by targeting miR-885-5p/RBBP4 axis. Cancer Cell Int. 2021 Jan 11;21(1):39. doi: 10.1186/s12935-020-01690-1. PMID: 33430870; PMCID: PMC7798264. Chen PY, Li XD, Ma WN, Li H, Li MM, Yang XY, Li SY. Comprehensive Transcriptomic Analysis and Experimental Validation Identify lncRNA HOXA-AS2/miR-184/COL6A2 as the Critical ceRNA Regulation Involved in Low-Grade Glioma Recurrence. Onco Targets Ther. 2020 Jun 3;13:4999-5016. doi: 10.2147/OTT.S245896. PMID: 32581558; PMCID: PMC7276213.
HOXA-AS3	Upregulated	Chen W, Li Q, Zhang G, Wang H, Zhu Z, Chen L. LncRNA HOXA-AS3 promotes the malignancy of glioblastoma through regulating miR-455-5p/USP3 axis. J Cell Mol Med. 2020 Oct;24(20):11755-11767. doi: 10.1111/jcmm.15788. Epub 2020 Sep 11. PMID: 32918360; PMCID: PMC7579690.
HOXB-AS1	Upregulated	Bi Y, Mao Y, Su Z, Du J, Ye L, Xu F. HOXB-AS1 accelerates the tumorigenesis of glioblastoma via modulation of HOBX2 and HOBX3 at transcriptional and posttranscriptional levels. J Cell Physiol. 2021 Jan;236(1):93-106. doi: 10.1002/jcp.29499. Epub 2020 Aug 16. PMID: 33459377.
HOXC-AS2	Upregulated	Dong N, Guo J, Han S, Bao L, Diao Y, Lin Z. Positive feedback loop of lncRNA HOXC-AS2/miR-876-5p/ZEB1 to regulate EMT in glioma. Onco Targets Ther. 2019 Sep 16;12:7601-7609. doi: 10.2147/OTT.S216134. PMID: 31571911; PMCID: PMC6754333.
HULC	Upregulated	Hu Y, Ye S, Li Q, Yin T, Wu J, He J. Quantitative Proteomics Analysis Indicates That Upregulation of lncRNA HULC Promotes Pathogenesis of Glioblastoma Cells. Onco Targets Ther. 2020 Jun 22;13:5927-5938. doi: 10.2147/OTT.S252915. PMID: 32606802; PMCID: PMC7319537.
KCNQ1OT1	Upregulated	Gong W, Zheng J, Liu X, et al. Knockdown of Long Non-Coding RNA KCNQ1OT1 Restrained Glioma Cells' Malignancy by Activating miR-370/CCNE2 Axis. Front Cell Neurosci. 2017;11:84. Published 2017 Mar 22. doi:10.3389/fncel.2017.00084 Ding P, Liang B, Shou J, Wang X. lncRNA KCNQ1OT1 promotes proliferation and invasion of glioma cells by targeting the miR-375/YAP pathway. Int J Mol Med. 2020 Dec;46(6):1983-1992. doi: 10.3892/ijmm.2020.4760. Epub 2020 Oct 19. PMID: 33125099; PMCID: PMC7595660.
KTN1-AS1	Upregulated	Mu Y, Tang Q, Feng H, Zhu L, Wang Y. lncRNA KTN1-AS1 promotes glioma cell proliferation and invasion by negatively regulating miR-505-3p.

		Oncol Rep. 2020 Dec;44(6):2645-2655. doi: 10.3892/or.2020.7821. Epub 2020 Oct 22. PMID: 33125151; PMCID: PMC7640367.
LEF1-AS1	Upregulated	Wang J, Liu X, Yan C, Liu J, Wang S, Hong Y, Gu A, Zhao P. LEF1-AS1, a long-noncoding RNA, promotes malignancy in glioblastoma. Onco Targets Ther. 2017 Aug 28;10:4251-4260. doi: 10.2147/OTT.S130365. PMID: 28894380; PMCID: PMC5584905.
LGMNP1	Upregulated	Liao K, Qian Z, Zhang S, Chen B, Li Z, Huang R, Cheng L, Wang T, Yang R, Lan J, Lu X, Kong L, Song X, Qiu Y, Lin Y. The LGMN pseudogene promotes tumor progression by acting as a miR-495-3p sponge in glioblastoma. Cancer Lett. 2020 Oct 10;490:111-123. doi: 10.1016/j.canlet.2020.07.012. Epub 2020 Jul 22. PMID: 32711096.
LINC-PINT	Downregulated	Zhu H, Chen Z, Shen L, Tang T, Yang M, Zheng X. Long Noncoding RNA LINC-PINT Suppresses Cell Proliferation, Invasion, and EMT by Blocking Wnt/β-Catenin Signaling in Glioblastoma. Front Pharmacol. 2021 Jan 11;11:586653. doi: 10.3389/fphar.2020.586653. PMID: 33505307; PMCID: PMC7832092.
LINC00115	Upregulated	Tang J, Yu B, Li Y, Zhang W, Alvarez AA, Hu B, Cheng SY, Feng H. TGF-β-activated lncRNA LINC00115 is a critical regulator of glioma stem-like cell tumorigenicity. EMBO Rep. 2019 Dec 5;20(12):e48170. doi: 10.15252/embr.201948170. Epub 2019 Oct 10. PMID: 31599491; PMCID: PMC6893290.
LINC00152	Upregulated	Cai J, Zhang J, Wu P, Yang W, Ye Q, Chen Q, Jiang C. Blocking LINC00152 suppresses glioblastoma malignancy by impairing mesenchymal phenotype through the miR-612/AKT2/NF-κB pathway. J Neurooncol. 2018 Nov;140(2):225-236. doi: 10.1007/s11060-018-2951-0. Epub 2018 Jul 24. PMID: 30043319.<br>Reon BJ, Takao Real Karia B, Kiran M, Dutta A. LINC00152 Promotes Invasion through a 3'-Hairpin Structure and Associates with Prognosis in Glioblastoma. Mol Cancer Res. 2018 Oct;16(10):1470-1482. doi: 10.1158/1541-7786.MCR-18-0322. Epub 2018 Jul 10. PMID: 29991527; PMCID: PMC6170721.
LINC00294	Downregulated	Zhou X, Lv L, Zhang Z, Wei S, Zheng T. LINC00294 negatively modulates cell proliferation in glioma through a neurofilament medium-mediated pathway via interacting with miR-1278. J Gene Med. 2020 Oct;22(10):e3235. doi: 10.1002/jgm.3235. Epub 2020 Jun 18. PMID: 32450002.
LINC00467	Upregulated	Liang R, Tang Y. LINC00467 knockdown repressed cell proliferation but stimulated cell apoptosis in glioblastoma via miR-339-3p/IP6K2 axis. Cancer Biomark. 2020;28(2):169-180. doi: 10.3233/CBM-190939. PMID: 32176627.
LINC00470	Upregulated	Liu C, Fu H, Liu X, Lei Q, Zhang Y, She X, Liu Q, Liu Q, Sun Y, Li G, Wu M. LINC00470 Coordinates the Epigenetic Regulation of ELFN2 to Distract GBM Cell Autophagy. Mol Ther. 2018 Sep 5;26(9):2267-2281. doi: 10.1016/j.ymthe.2018.06.019. Epub 2018 Jun 27. PMID: 30037656; PMCID: PMC6127511.<br>Liu C, Zhang Y, She X, Fan L, Li P, Feng J, Fu H, Liu Q, Liu Q, Zhao C, Sun Y, Wu M. A cytoplasmic long noncoding RNA LINC00470 as a new AKT activator to mediate glioblastoma cell autophagy. J Hematol Oncol. 2018 Jun 4;11(1):77. doi: 10.1186/s13045-018-0619-z. PMID: 29866190; PMCID: PMC5987392.
LINC00511	Upregulated	Du X, Tu Y, Liu S, Zhao P, Bao Z, Li C, Li J, Pan M, Ji J. LINC00511 contributes to glioblastoma tumorigenesis and epithelial-mesenchymal

		transition via LINC00511/miR-524-5p/YB1/ZEB1 positive feedback loop. J Cell Mol Med. 2020 Jan;24(2):1474-1487. doi: 10.1111/jcmm.14829. Epub 2019 Dec 19. PMID: 31856394; PMCID: PMC6991637.
LINC00599	Downregulated	Fu Q, Li S, Zhou Q, Yalikun K, Yisireyili D, Xia M. Low LINC00599 expression is a poor prognostic factor in glioma. Biosci Rep. 2019 Apr 2;39(4):BSR20190232. doi: 10.1042/BSR20190232. PMID: 30867254; PMCID: PMC6443953.
LINC00641	Downregulated	Yang J, Yu D, Liu X, Changyong E, Yu S. LINC00641/miR-4262/NRGN axis confines cell proliferation in glioma. Cancer Biol Ther. 2020 Aug 2;21(8):758-766. doi: 10.1080/15384047.2020.1776581. Epub 2020 Jun 16. PMID: 32543324; PMCID: PMC7515501.
LINC00645	Upregulated	Li C, Zheng H, Hou W, Bao H, Xiong J, Che W, Gu Y, Sun H, Liang P. Long non-coding RNA linc00645 promotes TGF-β-induced epithelial-mesenchymal transition by regulating miR-205-3p-ZEB1 axis in glioma. Cell Death Dis. 2019 Sep 26;10(10):717. doi: 10.1038/s41419-019-1948-8. PMID: 31558707; PMCID: PMC6763487.
LINC00998	Downregulated	Cai H, Yu Y, Ni X, Li C, Hu Y, Wang J, Chen F, Xi S, Chen Z. LncRNA LINC00998 inhibits the malignant glioma phenotype via the CBX3-mediated c-Met/Akt/mTOR axis. Cell Death Dis. 2020 Dec 2;11(12):1032. doi: 10.1038/s41419-020-03247-6. PMID: 33268783; PMCID: PMC7710718.
LINC01057	Upregulated	Tang G, Luo L, Zhang J, Zhai D, Huang D, Yin J, Zhou Q, Zhang Q, Zheng G. lncRNA LINC01057 promotes mesenchymal differentiation by activating NF-κB signaling in glioblastoma. Cancer Lett. 2021 Feb 1;498:152-164. doi: 10.1016/j.canlet.2020.10.047. Epub 2020 Oct 31. PMID: 33130316.
LINC01094	Upregulated	Li XX, Yu Q. Linc01094 Accelerates the Growth and Metastatic-Related Traits of Glioblastoma by Sponging miR-126-5p. Onco Targets Ther. 2020 Oct 6;13:9917-9928. doi: 10.2147/OTT.S263091. PMID: 33116576; PMCID: PMC7547807.
LINC01152	Upregulated	Wu J, Wang N, Yang Y, Jiang G, Mu Q, Zhan H, Li F. LINC01152 upregulates MAML2 expression to modulate the progression of glioblastoma multiforme via Notch signaling pathway. Cell Death Dis. 2021 Jan 22;12(1):115. doi: 10.1038/s41419-020-03163-9. PMID: 33483471; PMCID: PMC7822850.
LINC01198	Upregulated	Xie Y, Cheng Y. LINC01198 facilitates gliomagenesis through activating PI3K/AKT pathway. RNA Biol. 2020 Jul;17(7):1040-1052. doi: 10.1080/15476286.2020.1755112. Epub 2020 May 7. PMID: 32378450; PMCID: PMC7549703.
LINC01426	Upregulated	Cao J, Tang Z, Su Z. Long non-coding RNA LINC01426 facilitates glioblastoma progression via sponging miR-345-3p and upregulation of VAMP8. Cancer Cell Int. 2020 Jul 20;20:327. doi: 10.1186/s12935-020-01416-3. PMID: 32699526; PMCID: PMC7372762.
LINC01446	Upregulated	Zhang L, Wang Q, Wang F, Zhang X, Zhang L, Tang Y, Wang S. LncRNA LINC01446 promotes glioblastoma progression by modulating miR-489-3p/TPT1 axis. Biochem Biophys Res Commun. 2018 Sep 10;503(3):1484-1490. doi: 10.1016/j.bbrc.2018.07.067. Epub 2018 Jul 17. PMID: 30029885.
LINC01579	Upregulated	Chai Y, Xie M. LINC01579 promotes cell proliferation by acting as a ceRNA of miR-139-5p to upregulate EIF4G2 expression in glioblastoma. J Cell Physiol. 2019 Dec;234(12):23658-23666. doi: 10.1002/jcp.28933. Epub 2019 Jun 11. PMID: 31187495.
Lnc-TALC	Upregulated	Wu P, Cai J, Chen Q, Han B, Meng X, Li Y, Li Z, Wang R, Lin L, Duan C, Kang C, Jiang C. Lnc-TALC promotes O^6-methylguanine-DNA methyltransferase expression via regulating the c-Met pathway by competitively

		binding with miR-20b-3p. Nat Commun. 2019 May 3;10(1):2045. doi: 10.1038/s41467-019-10025-2. PMID: 31053733; PMCID: PMC6499807.
lncHERG	Upregulated	Shi J, Wang YJ, Sun CR, Qin B, Zhang Y, Chen G. Long noncoding RNA lncHERG promotes cell proliferation, migration and invasion in glioblastoma. Oncotarget. 2017 Nov 14;8(64):108031-108041. doi: 10.18632/oncotarget.22446. PMID: 29296221; PMCID: PMC5746123.
lncRNA-p21	Downregulated	Yang W, Yu H, Shen Y, Liu Y, Yang Z, Sun T. MiR-146b-5p overexpression attenuates stemness and radioresistance of glioma stem cells by targeting HuR/lincRNA-p21/β-catenin pathway. Oncotarget. 2016 Jul 5;7(27):41505-41526. doi: 10.18632/oncotarget.9214. PMID: 27166258; PMCID: PMC5173075.
LOXL1-AS1	Upregulated	Wang H, Li L, Yin L. Silencing LncRNA LOXL1-AS1 attenuates mesenchymal characteristics of glioblastoma via NF-κB pathway. Biochem Biophys Res Commun. 2018 Jun 2;500(2):518-524. doi: 10.1016/j.bbrc.2018.04.133. Epub 2018 Apr 21. PMID: 29678575.
MALAT1	Upregulated	Liao K, Lin Y, Gao W, Xiao Z, Medina R, Dmitriev P, Cui J, Zhuang Z, Zhao X, Qiu Y, Zhang X, Ge J, Guo L. Blocking lncRNA MALAT1/miR-199a/ZHX1 Axis Inhibits Glioblastoma Proliferation and Progression. Mol Ther Nucleic Acids. 2019 Dec 6;18:388-399. doi: 10.1016/j.omtn.2019.09.005. Epub 2019 Sep 17. PMID: 31648104; PMCID: PMC6819876.<br>Voce DJ, Bernal GM, Wu L, Crawley CD, Zhang W, Mansour NM, Cahill KE, Szymura SJ, Uppal A, Raleigh DR, Spretz R, Nunez L, Larsen G, Khodarev NN, Weichselbaum RR, Yamini B. Temozolomide Treatment Induces lncRNA MALAT1 in an NF-κB and p53 Codependent Manner in Glioblastoma. Cancer Res. 2019 May 15;79(10):2536-2548. doi: 10.1158/0008-5472.CAN-18-2170. Epub 2019 Apr 2. PMID: 30940658; PMCID: PMC6522287.<br>Cai T, Liu Y, Xiao J. Long noncoding RNA MALAT1 knockdown reverses chemoresistance to temozolomide via promoting microRNA-101 in glioblastoma. Cancer Med. 2018 Apr;7(4):1404-1415. doi: 10.1002/cam4.1384. Epub 2018 Feb 26. PMID: 29479863; PMCID: PMC5911628.<br>Kim SS, Harford JB, Moghe M, Rait A, Pirollo KF, Chang EH. Targeted nanocomplex carrying siRNA against MALAT1 sensitizes glioblastoma to temozolomide. Nucleic Acids Res. 2018 Feb 16;46(3):1424-1440. doi: 10.1093/nar/gkx1221. PMID: 29202181; PMCID: PMC5815062.<br>Chen W, Xu XK, Li JL, Kong KK, Li H, Chen C, He J, Wang F, Li P, Ge XS, Li FC. MALAT1 is a prognostic factor in glioblastoma multiforme and induces chemoresistance to temozolomide through suppressing miR-203 and promoting thymidylate synthase expression. Oncotarget. 2017 Apr 4;8(14):22783-22799. doi: 10.18632/oncotarget.15199. PMID: 28187000; PMCID: PMC5410262.
MATN-AS1	Downregulated	Han N, Yang L, Zhang X, Zhou Y, Chen R, Yu Y, Dong Z, Zhang M. LncRNA MATN1-AS1 prevents glioblastoma cell from proliferation and invasion via RELA regulation and MAPK signaling pathway. Ann Transl Med. 2019 Dec;7(23):784. doi: 10.21037/atm.2019.11.36. PMID: 32042800; PMCID: PMC6990012.
MCM3AP-AS1	Upregulated	Yang C, Zheng J, Xue Y, Yu H, Liu X, Ma J, Liu L, Wang P, Li Z, Cai H, Liu Y. The Effect of MCM3AP-AS1/miR-211/KLF5/AGGF1 Axis Regulating Glioblastoma Angiogenesis. Front Mol Neurosci. 2018 Jan 9;10:437. doi: 10.3389/fnmol.2017.00437. PMID: 29375300; PMCID: PMC5767169.
MEG3	Downregulated	Luo Q, Zhang F, Li W, Wang F, Wu L, Huang B. Overexpression of lncRNA MEG3 inhibits proliferation and invasion of glioblastoma U251

		cells in vitro by suppressing HIF1α expression. Nan Fang Yi Ke Da Xue Xue Bao. 2021 Jan 30;41(1):141-145. Chinese. doi: 10.12122/j.issn.1673-4254.2021.01.21. PMID: 33509767; PMCID: PMC7867485.<br>Gong X, Huang MY. Tumor-Suppressive Function of lncRNA-MEG3 in Glioma Cells by Regulating miR-6088/SMARCB1 Axis. Biomed Res Int. 2020 Jan 11;2020:4309161. doi: 10.1155/2020/4309161. PMID: 32420340; PMCID: PMC7201742.<br>Buccarelli M, Lulli V, Giuliani A, Signore M, Martini M, D'Alessandris QG, Giannetti S, Novelli A, Ilari R, Giurato G, Boe A, Castellani G, Spartano S, Marangi G, Biffoni M, Genuardi M, Pallini R, Marziali G, Ricci-Vitiani L. Deregulated expression of the imprinted DLK1-DIO3 region in glioblastoma stemlike cells: tumor suppressor role of lncRNA MEG3. Neuro Oncol. 2020 Dec 18;22(12):1771-1784. doi: 10.1093/neuonc/noaa127. PMID: 32459347; PMCID: PMC7746944.<br>Yang Z, Bian E, Xu Y, Ji X, Tang F, Ma C, Wang H, Zhao B. Meg3 Induces EMT and Invasion of Glioma Cells via Autophagy. Onco Targets Ther. 2020 Jan 31;13:989-1000. doi: 10.2147/OTT.S239648. PMID: 32099402; PMCID: PMC6999788.
miR143HG	Downregulated	Wang P, Bao W, Liu X, Xi W. LncRNA miR143HG inhibits the proliferation of glioblastoma cells by sponging miR-504. Int J Neurosci. 2021 Feb 2:1-9. doi: 10.1080/00207454.2020.1865950. Epub ahead of print. PMID: 33461388.
miR155HG	Upregulated	Wu W, Yu T, Wu Y, Tian W, Zhang J, Wang Y. The miR155HG/miR-185/ANXA2 loop contributes to glioblastoma growth and progression. J Exp Clin Cancer Res. 2019 Mar 21;38(1):133. doi: 10.1186/s13046-019-1132-0. PMID: 30898167; PMCID: PMC6427903.<br>Peng L, Chen Z, Chen Y, Wang X, Tang N. MIR155HG is a prognostic biomarker and associated with immune infiltration and immune checkpoint molecules expression in multiple cancers. Cancer Med. 2019 Dec;8(17):7161-7173. doi: 10.1002/cam4.2583. Epub 2019 Sep 30. PMID: 31568700; PMCID: PMC6885872.
miR4435-2HG	Upregulated	Xu H, Zhang B, Yang Y, Li Z, Zhao P, Wu W, Zhang H, Mao J. LncRNA MIR4435-2HG potentiates the proliferation and invasion of glioblastoma cells via modulating miR-1224-5p/TGFBR2 axis. J Cell Mol Med. 2020 Jun;24(11):6362-6372. doi: 10.1111/jcmm.15280. Epub 2020 Apr 22. PMID: 32319715; PMCID: PMC7294147.
MNX1-AS1	Upregulated	Gao Y, Xu Y, Wang J, Yang X, Wen L, Feng J. lncRNA MNX1-AS1 Promotes Glioblastoma Progression Through Inhibition of miR-4443. Oncol Res. 2019 Feb 21;27(3):341-347. doi: 10.3727/096504018X15228909735079. Epub 2018 Apr 20. PMID: 29678219; PMCID: PMC7848264.
MVIH	Downregulated	Cardoso AM, Morais CM, Rebelo O, Tão H, Barbosa M, Pedroso de Lima MC, Jurado AS. Downregulation of long non-protein coding RNA MVIH impairs glioblastoma cell proliferation and invasion through a miR-302a-dependent mechanism. Hum Mol Genet. 2021 Jan 12:ddab009. doi: 10.1093/hmg/ddab009. Epub ahead of print. PMID: 33438023.
NEAT1	Upregulated	Gao XY, Zang J, Zheng MH, Zhang YF, Yue KY, Cao XL, Cao Y, Li XX, Han H, Jiang XF, Liang L. Temozolomide Treatment Induces HMGB1 to Promote the Formation of Glioma Stem Cells via the TLR2/NEAT1/Wnt Pathway in Glioblastoma. Front Cell Dev Biol. 2021 Feb 1;9:620883. doi: 10.3389/fcell.2021.620883. PMID: 33614649; PMCID: PMC7891666.<br>Bi CL, Liu JF, Zhang MY, Lan S, Yang ZY, Fang JS. LncRNA NEAT1 promotes malignant phenotypes and TMZ resistance in glioblastoma stem

		cells by regulating let-7g-5p/MAP3K1 axis. Biosci Rep. 2020 Oct 30;40(10):BSR20201111. doi: 10.1042/BSR20201111. PMID: 33057597; PMCID: PMC7601351.<br>Lulli V, Buccarelli M, Ilari R, Castellani G, De Dominicis C, Di Giamberardino A, D Alessandris QG, Giannetti S, Martini M, Stumpo V, Boe A, De Luca G, Biffoni M, Marziali G, Pallini R, Ricci-Vitiani L. Mir-370-3p Impairs Glioblastoma Stem-Like Cell Malignancy Regulating a Complex Interplay between HMGA2/HIF1A and the Oncogenic Long Non-Coding RNA (lncRNA) NEAT1. Int J Mol Sci. 2020 May 20;21(10):3610. doi: 10.3390/ijms21103610. PMID: 32443824; PMCID: PMC7279259.<br>Chen Q, Cai J, Wang Q, Wang Y, Liu M, Yang J, Zhou J, Kang C, Li M, Jiang C. Long Noncoding RNA NEAT1, Regulated by the EGFR Pathway, Contributes to Glioblastoma Progression Through the WNT/β-Catenin Pathway by Scaffolding EZH2. Clin Cancer Res. 2018 Feb 1;24(3):684-695. doi: 10.1158/1078-0432.CCR-17-0605. Epub 2017 Nov 14. PMID: 29138341.
PCAT6	Upregulated	Liu P, Zhao P, Li B, Xu D, Wang K. LncRNA PCAT6 Regulated by YY1 Accelerates the Progression of Glioblastoma via miR-513/IGF2BP1. Neurochem Res. 2020 Dec;45(12):2894-2902. doi: 10.1007/s11064-020-03138-4. Epub 2020 Sep 29. PMID: 32990800.
PCED1B-AS1	Upregulated	Yao Z, Zhang Q, Guo F, Guo S, Yang B, Liu B, Li P, Li J, Guan S, Liu X. Long Noncoding RNA PCED1B-AS1 Promotes the Warburg Effect and Tumorigenesis by Upregulating HIF-1α in Glioblastoma. Cell Transplant. 2020 Jan-Dec;29:963689720906777. doi: 10.1177/0963689720906777. PMID: 32326742; PMCID: PMC7444212.
PSMB8-AS1	Upregulated	Hu T, Wang F, Han G. LncRNA PSMB8-AS1 acts as ceRNA of miR-22-3p to regulate DDIT4 expression in glioblastoma. Neurosci Lett. 2020 May 29;728:134896. doi: 10.1016/j.neulet.2020.134896. Epub 2020 Mar 6. PMID: 32151711.
PSMG3-AS1	Upregulated	Chen L, Wang G, Xu Z, Lin K, Mu S, Pan Y, Shan M. Overexpression of LncRNA PSMG3-AS1 Distinguishes Glioblastomas from Sarcoidosis. J Mol Neurosci. 2020 Dec;70(12):2015-2019. doi: 10.1007/s12031-020-01605-9. Epub 2020 Jun 11. PMID: 32529538.
PVT1	Upregulated	Jin Z, Piao LH, Sun GC, Lv CX, Jing Y, Jin RH. Long non-coding RNA plasmacytoma variant translocation 1 (PVT1) promotes glioblastoma multiforme progression via regulating miR-1301-3p/TMBIM6 axis. Eur Rev Med Pharmacol Sci. 2020 Nov;24(22):11658-11665. doi: 10.26355/eurrev_202011_23810. PMID: 33275233.
PWRN1	Downregulated	Jiang J, Wang X, Lu J. PWRN1 Suppressed Cancer Cell Proliferation and Migration in Glioblastoma by Inversely Regulating hsa-miR-21-5p. Cancer Manag Res. 2020 Jul 2;12:5313-5322. doi: 10.2147/CMAR.S250166. PMID: 32753949; PMCID: PMC7342408.
PXN-AS1	Upregulated	Chen H, Hou G, Yang J, Chen W, Guo L, Mao Q, Ge J, Zhang X. SOX9-activated PXN-AS1 promotes the tumorigenesis of glioblastoma by EZH2-mediated methylation of DKK1. J Cell Mol Med. 2020 Jun;24(11):6070-6082. doi: 10.1111/jcmm.15189. Epub 2020 Apr 23. PMID: 32329150; PMCID: PMC7294137.
RAD21	Upregulated	Mao Y, Shen G, Su Z, Du J, Xu F, Yu Y. RAD21 inhibited transcription of tumor suppressor MIR4697HG and led to glioma tumorigenesis. Biomed Pharmacother. 2020 Mar;123:109759. doi: 10.1016/j.biopha.2019.109759. Epub 2019 Dec 26. PMID: 31884342.
RMST	Upregulated	Liu C, Peng Z, Li P, Fu H, Feng J, Zhang Y, Liu T, Liu Y, Liu Q, Liu Q, Li D, Wu M. lncRNA RMST Suppressed GBM Cell Mitophagy through

		Enhancing FUS SUMOylation. Mol Ther Nucleic Acids. 2020 Mar 6;19:1198-1208. doi: 10.1016/j.omtn.2020.01.008. Epub 2020 Jan 18. PMID: 32069702; PMCID: PMC7019048.
RNCR3	Downregulated	Zhang L, Cao Y, Wei M, Jiang X, Jia D. Long noncoding RNA-RNCR3 overexpression deleteriously affects the growth of glioblastoma cells through miR-185-5p/Krüppel-like factor 16 axis. J Cell Biochem. 2018 Nov;119(11):9081-9089. doi: 10.1002/jcb.27167. Epub 2018 Jun 28. PMID: 29953649.
RP11-838N2.4	Downregulated	Liu Y, Xu N, Liu B, Huang Y, Zeng H, Yang Z, He Z, Guo H. Long noncoding RNA RP11-838N2.4 enhances the cytotoxic effects of temozolomide by inhibiting the functions of miR-10a in glioblastoma cell lines. Oncotarget. 2016 Jul 12;7(28):43835-43851. doi: 10.18632/oncotarget.9699. PMID: 27270310; PMCID: PMC5190063.
RPSAP52	Upregulated	Wang S, Guo X, Lv W, Li Y, Zhang L, Dong C, Zhang J, Cheng G. LncRNA RPSAP52 Upregulates TGF-β1 to Increase Cancer Cell Stemness and Predict Postoperative Survival in Glioblastoma. Cancer Manag Res. 2020 Apr 15;12:2541-2547. doi: 10.2147/CMAR.S227496. PMID: 32368136; PMCID: PMC7170709.
RUNX1-IT1	Upregulated	Wu Z. MiR-195 connects lncRNA RUNX1-IT1 and cyclin D1 to regulate the proliferation of glioblastoma cells. Int J Neurosci. 2021 Feb 3:1-6. doi: 10.1080/00207454.2021.1881090. Epub ahead of print. PMID: 33507136.
SAMMSON	Upregulated	Ni H, Wang K, Xie P, Zuo J, Liu W, Liu C. LncRNA SAMMSON Knockdown Inhibits the Malignancy of Glioblastoma Cells by Inactivation of the PI3K/Akt Pathway. Cell Mol Neurobiol. 2021 Jan;41(1):79-90. doi: 10.1007/s10571-020-00833-2. Epub 2020 Mar 31. PMID: 32236901.
SBF2-AS1	Upregulated	Zhang Z, Yin J, Lu C, Wei Y, Zeng A, You Y. Exosomal transfer of long non-coding RNA SBF2-AS1 enhances chemoresistance to temozolomide in glioblastoma. J Exp Clin Cancer Res. 2019 Apr 16;38(1):166. doi: 10.1186/s13046-019-1139-6. PMID: 30992025; PMCID: PMC6469146.<br>Yu H, Zheng J, Liu X, Xue Y, Shen S, Zhao L, Li Z, Liu Y. Transcription Factor NFAT5 Promotes Glioblastoma Cell-driven Angiogenesis via SBF2-AS1/miR-338-3p-Mediated EGFL7 Expression Change. Front Mol Neurosci. 2017 Sep 21;10:301. doi: 10.3389/fnmol.2017.00301. PMID: 28983240; PMCID: PMC5613209.
SChLAP1	Upregulated	Ji J, Xu R, Ding K, Bao G, Zhang X, Huang B, Wang X, Martinez A, Wang X, Li G, Miletic H, Thorsen F, Bjerkvig R, Xiang L, Han B, Chen A, Li X, Wang J. Long Noncoding RNA *SChLAP1* Forms a Growth-Promoting Complex with HNRNPL in Human Glioblastoma through Stabilization of ACTN4 and Activation of NF-κB Signaling. Clin Cancer Res. 2019 Nov 15;25(22):6868-6881. doi: 10.1158/1078-0432.CCR-19-0747. Epub 2019 Sep 6. PMID: 31492748.
SLC16A1-AS1	Upregulated	Long Y, Li H, Jin Z, Zhang X. LncRNA SLC16A1-AS1 is Upregulated in Glioblastoma and Promotes Cancer Cell Proliferation by Regulating miR-149 Methylation. Cancer Manag Res. 2021 Feb 10;13:1215-1223. doi: 10.2147/CMAR.S264613. PMID: 33603467; PMCID: PMC7882451.
SNHG1	Upregulated	Mi S, Du J, Liu J, Hou K, Ji H, Ma S, Ba Y, Chen L, Xie R, Hu S. FtMt promotes glioma tumorigenesis and angiogenesis via lncRNA SNHG1/miR-9-5p axis. Cell Signal. 2020 Nov;75:109749. doi: 10.1016/j.cellsig.2020.109749. Epub 2020 Aug 25. PMID: 32858123.
SNHG12	Upregulated	Lu C, Wei Y, Wang X, Zhang Z, Yin J, Li W, Chen L, Lyu X, Shi Z, Yan W, You Y. DNA-methylation-mediated activating of lncRNA SNHG12 promotes temozolomide resistance in glioblastoma. Mol Cancer. 2020 Feb

		10;19(1):28. doi: 10.1186/s12943-020-1137-5. PMID: 32039732; PMCID: PMC7011291.
SNHG14	Upregulated	Lu J, Liu X, Zheng J, Song J, Liu Y, Ruan X, Shen S, Shao L, Yang C, Wang D, Cai H, Cao S, Xue Y. Lin28A promotes IRF6-regulated aerobic glycolysis in glioma cells by stabilizing SNHG14. Cell Death Dis. 2020 Jun 11;11(6):447. doi: 10.1038/s41419-020-2650-6. PMID: 32527996; PMCID: PMC7289837.<br>Wang Q, Teng Y, Wang R, et al. The long non-coding RNA SNHG14 inhibits cell proliferation and invasion and promotes apoptosis by sponging miR-92a-3p in glioma. Oncotarget. 2018;9(15):12112-12124. Published 2018 Jan 4. doi:10.18632/oncotarget.23960
SNHG15	Upregulated	Li Z, Zhang J, Zheng H, Li C, Xiong J, Wang W, Bao H, Jin H, Liang P. Modulating lncRNA SNHG15/CDK6/miR-627 circuit by palbociclib, overcomes temozolomide resistance and reduces M2-polarization of glioma associated microglia in glioblastoma multiforme. J Exp Clin Cancer Res. 2019 Aug 28;38(1):380. doi: 10.1186/s13046-019-1371-0. PMID: 31462285; PMCID: PMC6714301.
SNHG4	Upregulated	Wang X, Tian W, Wu L, Wei Z, Li W, Xu Y, Li Y. LncRNA SNHG4 regulates miR-138/c-Met axis to promote the proliferation of glioblastoma cells. Neuroreport. 2020 Jun 7;31(9):657-662. doi: 10.1097/WNR.0000000000001469. PMID: 32427712.
SNHG5	Upregulated	Chen L, Gong X, Huang M. YY1-Activated Long Noncoding RNA SNHG5 Promotes Glioblastoma Cell Proliferation Through p38/MAPK Signaling Pathway. Cancer Biother Radiopharm. 2019 Nov;34(9):589-596. doi: 10.1089/cbr.2019.2779. Epub 2019 Oct 29. PMID: 31657621.
SNHG7	Upregulated	Chen Y, Yuan S, Ning T, Xu H, Guan B. SNHG7 Facilitates Glioblastoma Progression by Functioning as a Molecular Sponge for MicroRNA-449b-5p and Thereby Increasing MYCN Expression. Technol Cancer Res Treat. 2020 Jan-Dec;19:1533033820945802. doi: 10.1177/1533033820945802. PMID: 32720593; PMCID: PMC7388098.<br>Ren J, Yang Y, Xue J, Xi Z, Hu L, Pan SJ, Sun Q. Long noncoding RNA SNHG7 promotes the progression and growth of glioblastoma via inhibition of miR-5095. Biochem Biophys Res Commun. 2018 Feb 5;496(2):712-718. doi: 10.1016/j.bbrc.2018.01.109. PMID: 29360452.
SNORD76	Downregulated	Chen L, Han L, Wei J, Zhang K, Shi Z, Duan R, Li S, Zhou X, Pu P, Zhang J, Kang C. SNORD76, a box C/D snoRNA, acts as a tumor suppressor in glioblastoma. Sci Rep. 2015 Feb 26;5:8588. doi: 10.1038/srep08588. PMID: 25715874; PMCID: PMC5390076.
SOX2OT	Upregulated	Liu B, Zhou J, Wang C, Chi Y, Wei Q, Fu Z, Lian C, Huang Q, Liao C, Yang Z, Zeng H, Xu N, Guo H. LncRNA SOX2OT promotes temozolomide resistance by elevating SOX2 expression via ALKBH5-mediated epigenetic regulation in glioblastoma. Cell Death Dis. 2020 May 21;11(5):384. doi: 10.1038/s41419-020-2540-y. PMID: 32439916; PMCID: PMC7242335.
TALNEC2	Upregulated	Brodie S, Lee HK, Jiang W, Cazacu S, Xiang C, Poisson LM, Datta I, Kalkanis S, Ginsberg D, Brodie C. The novel long non-coding RNA TALNEC2, regulates tumor cell growth and the stemness and radiation response of glioma stem cells. Oncotarget. 2017 May 9;8(19):31785-31801. doi: 10.18632/oncotarget.15991. PMID: 28423669; PMCID: PMC5458248.
TCONS_00020456	Downregulated	Tang C, Wang Y, Zhang L, Wang J, Wang W, Han X, Mu C, Gao D. Identification of novel LncRNA targeting Smad2/PKCα signal pathway to negatively regulate malignant progression of glioblastoma. J Cell Physiol. 2020

		Apr;235(4):3835-3848. doi: 10.1002/jcp.29278. Epub 2019 Oct 11. PMID: 31603255; PMCID: PMC6972644.
TP73-AS1	Upregulated	Mazor G, Levin L, Picard D, Ahmadov U, Carén H, Borkhardt A, Reifenberger G, Leprivier G, Remke M, Rotblat B. The lncRNA TP73-AS1 is linked to aggressiveness in glioblastoma and promotes temozolomide resistance in glioblastoma cancer stem cells. Cell Death Dis. 2019 Mar 13;10(3):246. doi: 10.1038/s41419-019-1477-5. PMID: 30867410; PMCID: PMC6416247.
TPT1-AS1	Upregulated	Gao X, Cao Y, Li J, Wang C, He H. LncRNA TPT1-AS1 Sponges miR-23a-5p in Glioblastoma to Promote Cancer Cell Proliferation. Cancer Biother Radiopharm. 2020 Aug 12. doi: 10.1089/cbr.2019.3484. Epub ahead of print. PMID: 32783743.
TUSC7	Downregulated	Shang C, Tang W, Pan C, Hu X, Hong Y. Long non-coding RNA TUSC7 inhibits temozolomide resistance by targeting miR-10a in glioblastoma. Cancer Chemother Pharmacol. 2018 Apr;81(4):671-678. doi: 10.1007/s00280-018-3522-y. Epub 2018 Feb 3. PMID: 29397407.
UBA6-AS1	Upregulated	Cheng F, Liu J, Zhang Y, You Q, Chen B, Cheng J, Deng C. Long Non-Coding RNA UBA6-AS1 Promotes the Malignant Properties of Glioblastoma by Competitively Binding to microRNA-760 and Enhancing Homeobox A2 Expression. Cancer Manag Res. 2021 Jan 14;13:379-392. doi: 10.2147/CMAR.S287676. PMID: 33469379; PMCID: PMC7813458.
UBE2R2-AS1	Downregulated	Xu W, Hu GQ, Da Costa C, Tang JH, Li QR, Du L, Pan YW, Lv SQ. Long noncoding RNA UBE2R2-AS1 promotes glioma cell apoptosis via targeting the miR-877-3p/TLR4 axis. Onco Targets Ther. 2019 May 7;12:3467-3480. doi: 10.2147/OTT.S201732. PMID: 31123407; PMCID: PMC6511244.
Unigene56159	Upregulated	Jiang G, Dong H, Dong Y, Yang X. Long non-coding RNA Unigene56159 promotes glioblastoma multiforme cell proliferation and invasion through negatively regulating microRNA-194-5p. Mol Med Rep. 2020 Feb;21(2):768-776. doi: 10.3892/mmr.2019.10852. Epub 2019 Nov 26. PMID: 31789416; PMCID: PMC6947890.
VIM-AS1	Upregulated	Suo ST, Gong P, Peng XJ, Niu D, Guo YT. Knockdown of long non-coding RNA VIM-AS1 inhibits glioma cell proliferation and migration, and increases the cell apoptosis via modulation of WEE1 targeted by miR-105-5p. Eur Rev Med Pharmacol Sci. 2020 Jun;24(12):6834-6847. doi: 10.26355/eurrev_202006_21673. PMID: 32633376.
WDFY3-AS2	Downregulated	Wu F, Zhao Z, Chai R, Liu Y, Wang K, Wang Z, Li G, Huang R, Jiang H, Zhang K. Expression profile analysis of antisense long non-coding RNA identifies WDFY3-AS2 as a prognostic biomarker in diffuse glioma. Cancer Cell Int. 2018 Jul 28;18:107. doi: 10.1186/s12935-018-0603-2. PMID: 30069164; PMCID: PMC6064140.
WEE2-AS1	Upregulated	Lin H, Zuo D, He J, Ji T, Wang J, Jiang T. Long noncoding RNA WEE2-AS1 plays an oncogenic role in glioblastoma by functioning as a molecular sponge for microRNA-520f-3p. Oncol Res. 2020 Aug 24. doi: 10.3727/096504020X15982623243955. Epub ahead of print. PMID: 32838835.
XIST	Upregulated	Li HL, Han PH, Pan DQ, Chen G, Lu XH, Li J. LncRNA XIST regulates cell proliferation, migration and invasion of glioblastoma via regulating miR-448 and ROCK1. J Biol Regul Homeost Agents. 2020 Nov-Dec;34(6):2049-2058. doi: 10.23812/20-558-L. PMID: 33325210.<br>Gong M, Wang X, Mu L, Wang Y, Pan J, Yuan X, Zhou H, Xing J, Wang R, Sun J, Liu Q, Zhang X, Wang L, Chen Y, Pei Y, Li S, Liu L, Zhao Y, Yuan Y. SRC-1 enhances the stemness of glioblastoma by activating lncRNA XIST/

miR-152/ KLF4 pathway. Cancer Sci. 2020 Oct 14. doi: 10.1111/cas.14685. Epub ahead of print. PMID: 33090636.
Yao Y, Ma J, Xue Y, Wang P, Li Z, Liu J, Chen L, Xi Z, Teng H, Wang Z, Li Z, Liu Y. Knockdown of long non-coding RNA XIST exerts tumor-suppressive functions in human glioblastoma stem cells by up-regulating miR-152. Cancer Lett. 2015 Apr 1;359(1):75-86. doi: 10.1016/j.canlet.2014.12.051. Epub 2015 Jan 8. PMID: 25578780.
